# Modest Offset Difference Internuclear Selective Transfer via Homonuclear Dipolar Coupling

**DOI:** 10.1021/acs.jpclett.1c03871

**Published:** 2022-02-08

**Authors:** Evgeny Nimerovsky, Eszter E. Najbauer, Kumar Tekwani Movellan, Kai Xue, Stefan Becker, Loren B. Andreas

**Affiliations:** Department of NMR Based Structural Biology, Max Planck Institute for Biophysical Chemistry, Am Fassberg 11, 37077 Göttingen, Germany

## Abstract

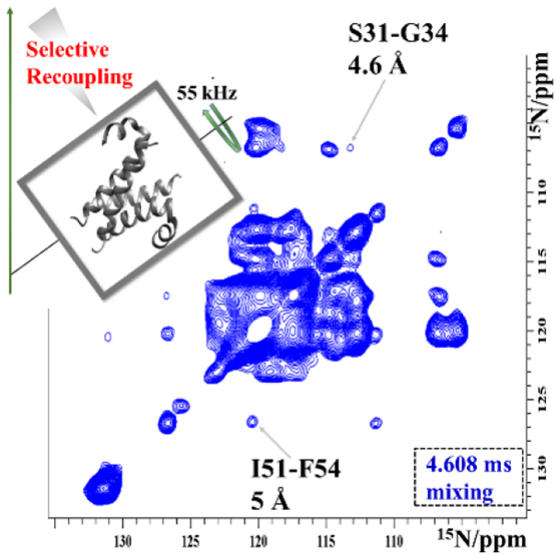

Homonuclear dipolar recoupling is routinely used for magic-angle spinning NMR-based structure determination. In fully protonated samples, only short proton–proton distances are accessible to broadband recoupling approaches because of high proton density. Selective methods allow detection of longer distances by directing polarization to a subset of spins. Here we introduce the selective pulse sequence MODIST, which recouples spins that have a modest chemical shift offset difference, and demonstrate it to selectively record correlations between amide protons. The sequence was selected for good retention of total signal, leading to up to twice the intensity for proton–proton correlations compared with other selective methods. The sequence is effective across a range of spinning conditions and magnetic fields, here tested at 55.555 and 100 kHz magic-angle spinning and at proton Larmor frequencies from 600 to 1200 MHz. For influenza A M2 in lipid bilayers, cross-peaks characteristic of a helical conformation are observed.

Proton-detected magic-angle spinning (MAS) NMR spectroscopy can be used to determine the structure and dynamics of proteins with atomic resolution. Proton–proton correlations obtained by recoupling homonuclear dipolar interactions are direct indicators of the protein fold.^1−11^ One broadly used method for dipolar recoupling is radio-frequency-driven recoupling (RFDR), first introduced by Gullion and Vega^12^ and Bennett et al.^13^ for moderate MAS rates and ^13^C recoupling. It is also applicable at ultrafast MAS rates of 55–100 kHz and above, where the effects of finite pulses become important^14^ and a heteronuclear version of the sequence becomes possible.^15^ Proton–proton RFDR has been widely applied for protein structure determination,^14,16−23^ which, for fully protonated proteins, is done by measuring a dense network of distances including side-chain protons.

A characteristic of broadband proton–proton recoupling in fully protonated samples is that only particularly close spins show correlations, while longer distances are hardly detectable.^24^ This is the consequence of high proton density in fully protonated protein samples and forms the basis for structure determination involving side-chain protons.^17^ Measurement of longer proton distances is challenging in these samples, even with second-order recoupling schemes^25−30^ that have been widely applied at both low and high MAS rates to correlate carbon and nitrogen spins.

Detection of longer distances is elegantly achieved by selective spin-labeling of ^13^C^31−33^ or ^1^H.^34−39^ For example, Linser et al.^39^ reported amide proton–proton correlations up to 10 Å for a perdeuterated microcrystalline sample. Using deuteration and specific methyl proton labeling, Huber et al.^40^ detected ^1^H–^1^H correlations for distances up to 6 Å. The former implemented broadband zero-quantum recoupling using RFDR, while the latter applied a double-quantum sequence, dipolar recoupling enhanced by amplitude modulation (DREAM).^41^ However, selective labeling is not always straightforward, in particular for membrane proteins, for which amide exchange may be inhibited.^42^

Long-distance proton–proton correlations can also be measured by using selective recoupling experiments.^43−50^ In band-selective spectral spin diffusion (BASS-SD)^47^ selective ^1^H–^1^H transfer occurs during a spin-lock pulse,^51^ while in selective phase-optimized recoupling (SPR)^46^ selective ^1^H–^1^H transfers occur between spins with symmetrical frequency offsets^46^ (*f*_A_ = – *f*_B_, where *f*_A_ and *f*_B_ are the offsets of protons A and B). Both methods show significant enhancement of the transferred signals with respect to RFDR. Xiao et al. recently published theoretical investigations of SPR pulses at low MAS rates.^52^ In particular, they investigated the behavior of *p*-SPR5 pulses at different flip angles, *p* = π/4, π/2, and 3π/4, and concluded that small flip angles result in a narrow selective bandwidth.

Here, we present a zero-quantum homonuclear dipolar recoupling method, the modest offset difference internuclear selective transfer (MODIST) pulse sequence, where selective transfer occurs between spins with small differences in their offsets. We based the MODIST sequence on the jump-return^53,54^ elements of SPR pulses^46^ and modified the phase, the flip angles, and the number of pulses in the block to maximize transfer between amide spins, minimize transfer between amide and aliphatic spins, and, crucially, retain maximal total amide signal. MODIST is constructed similarly to π/4-SPR4_2_, but the modified phase cycling significantly modifies its transfer characteristics. The MODIST block consists of 16 π/4-pulses with the following phase cycling: *yy̅x̅xx̅xy̅yy̅yxx̅xx̅yy̅* (Figure 1A). The total length of the sequence corresponds to four rotor periods, such that each pulse occupies one-quarter of the rotor period, with an rf-field power of half the MAS rate.

**Figure 1:**
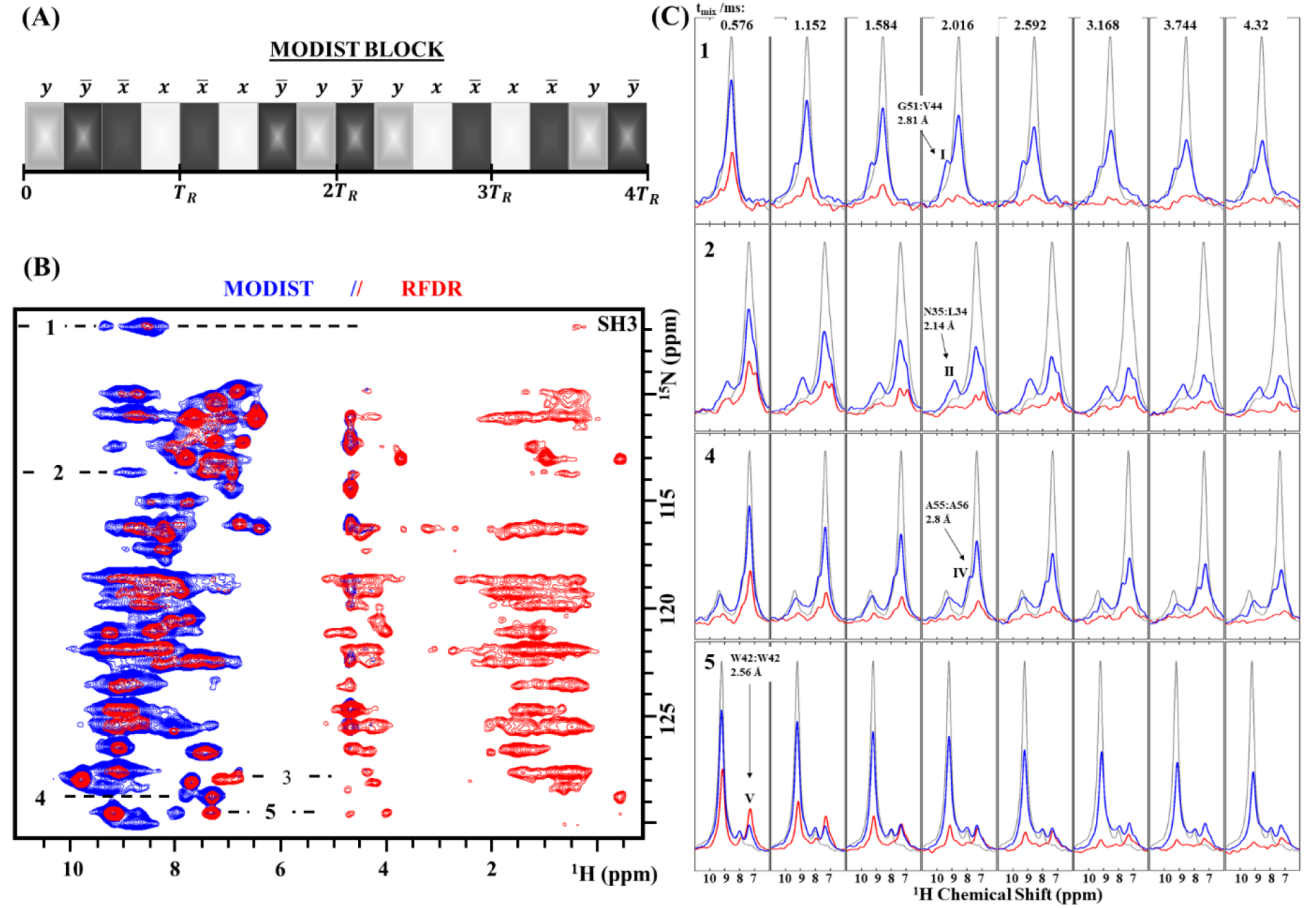
Comparison of RFDR and MODIST transfers in 2D (H)N(H)H spectra of microcrystalline SH3. (A) The MODIST pulse sequence −16 π/4 pulses with the phase cycle *yy̅x̅xx̅xy̅yy̅yxx̅xx̅yy̅* occupy four rotor periods (*T*_R_). (B) (H)N(H)H^MODIST^ (blue) and (H)N(H)H^RFDR^ (red) spectra (1.152 ms mixing). (C) Four slices from (H)N(H)H^RFDR^ (red) and (H)N(H)H^MODIST^ (blue) spectra, recorded at eight different mixing times (in ms): 0.576, 1.152, 1.584, 2.016, 2.592, 3.168, 3.744, and 4.32, as labeled. The chemical shifts of peaks I–V are (^15^N in ppm/^1^H in ppm): I, (106.8/9.35); II, (113.7/8.74); IV, (128.7/7.8); V, (129.4/7.34). The (H)NH reference spectrum is shown in gray. The proton carrier was set to 8.2 ppm for the mixing. Data were recorded from an 800 MHz spectrometer with 55.555 kHz MAS. XY8 phase cycling was used for RFDR. The full experimental details are given in the Supporting Information.

Numerical simulations of MODIST and comparison with SPR can be found in Figures S1–S10 of the Supporting Information, where we investigate the efficiency of the method assuming different values of dipolar coupling constants, offset differences, flip angles of the selective pulses, carrier frequency settings, phase cycle schemes, chemical shift anisotropy values, and MAS rates. In simulations (two amide and two aliphatic proton spins), the ratio between transferred and untransferred signals is inferior to SPR5_4_, but the total transfer efficiency of MODIST is better overall due to the high retention of the total amide signals. Although the transfer efficiency of MODIST pulses is much less dependent on the position of the proton carrier frequency in comparison to other selective methods, the position of the carrier has an influence on the width of the selective transfer, Δ*f*_MODIST_. We define Δ*f*_MODIST_ as the offset difference with which the transferred signal reaches at least 50% of the maximal transfer with respect to the signal with zero offset difference. On the basis of simulations, Δ*f*_MODIST_ of amide protons is ∼0.64 kHz (Figure S1C), when the position of the carrier is in the amide region (8.2 ppm). However, it can be increased up to ∼0.9 kHz by setting the carrier to the aliphatic region (Figure S6 and Table S1) without loss of efficiency.

MODIST selectively transfers signals at both 55 kHz (Figure 1) and 100 kHz MAS (Figure S10). Figure 1 compares MODIST with an efficient broadband recoupling method, RFDR, for fully protonated SH3. The MODIST implementation of the (H)N(H)H experiment, (H)N(H)H^MODIST^, shows a higher number of amide–amide correlations than (H)N(H)H^RFDR^ even with a short mixing of 1.152 ms (Figure 1B). While broadband RFDR recoupling predictably mixes signal among amide and aliphatic protons, MODIST results in minimal signal in the aliphatic region between 0 and 6 ppm. The buildup of selected peaks as a function of mixing time is shown in Figure 1C. Figures S11 and S12 compare XY4^1^_4_ and XY8 phase cycles for RFDR pulses as a function of mixing time.^55^Table 1 summarizes the assignments of isolated peaks (indexed as I, II, IV, and V), the corresponding distances, and the ^1^H–^1^H offset differences.

**Table 1:** Assignments, Distances (Proton–Proton), and ^1^H–^1^H Offset Differences of Selected Peaks from Figure 1^a^

		H–H distance (Å)	^1^H–^1^H offset diff (ppm)
G51 H^N^–V44 H^N^	I	2.81	0.82
N35 H^N^–L34 H^N^	II	2.07	1.41
W41 H^Nε1^–W41 H^δ1^	III	2.60	2.8
A55 H^N^–A56 H^N^	IV	2.8	0.51
W42 H^Nε1^–W42 H^δ1^	V	2.56	1.87

a Distances were taken from the crystal structure of the SH3 domain (PDB: 2NUZ).

While peaks I, II, and IV cannot be distinguished from the noise when using RFDR, they are above noise in the MODIST spectrum. However, for peak V, RFDR results in twice the transfer efficiency, and peak III (Figure 1B) is lower than the noise level for MODIST. Peaks III and V are intra-side-chain correlations between protons of W41 and W42 indole, respectively. The low MODIST signal for these peaks is explained by the comparably large offset difference between aromatic H^Nε1^ and H^δ1^ proton spins, which is 1.87 and 2.8 ppm (or 1.5 and 2.24 kHz at an 800 MHz spectrometer) for W42 (peak V) and W41 (peak III), respectively. The intensity of peaks I–III is retained at relatively long MODIST mixing time (Figure 1C), which again emphasizes that MODIST retains total signal during mixing.

To resolve long-distance correlations and to test the method at higher magnetic field where the amide frequency range is increased, we recorded a 3D (H)N(H)(H)NH^MODIST^ spectrum with 6.48 ms mixing at a 1200 MHz spectrometer. The proton carrier frequency was set to 3 ppm. Figure 2 shows the ^15^N–^15^N projection with the assignment of selected peaks based on the chemical shifts.^34,56,57^ With 6.48 ms mixing, we detect seven peaks correlated to G51, with the longest distance at 6.8 Å (G51-K43). The longest assigned distance is 7.3 Å, between D14 and M25. Peaks corresponding to these long distances likely arise due to significant contribution of relayed transfer rather than direct transfer alone. From the SH3 structure, it is evident that direct transfer is detected for distances of at least 4.5 Å (Figure 2). We also recorded a 3D (H)N(H)(H)NH^MODIST^ spectrum with 2.016 ms mixing using an 800 MHz spectrometer. Although numerous correlations were observed in the spectrum, most belong to nuclei within 4.5 Å due to a short mixing of 2.016 ms. This spectrum is displayed in Figure S13A. Additional assignments of Figure 2 are displayed in Figure S13B.

**Figure 2:**
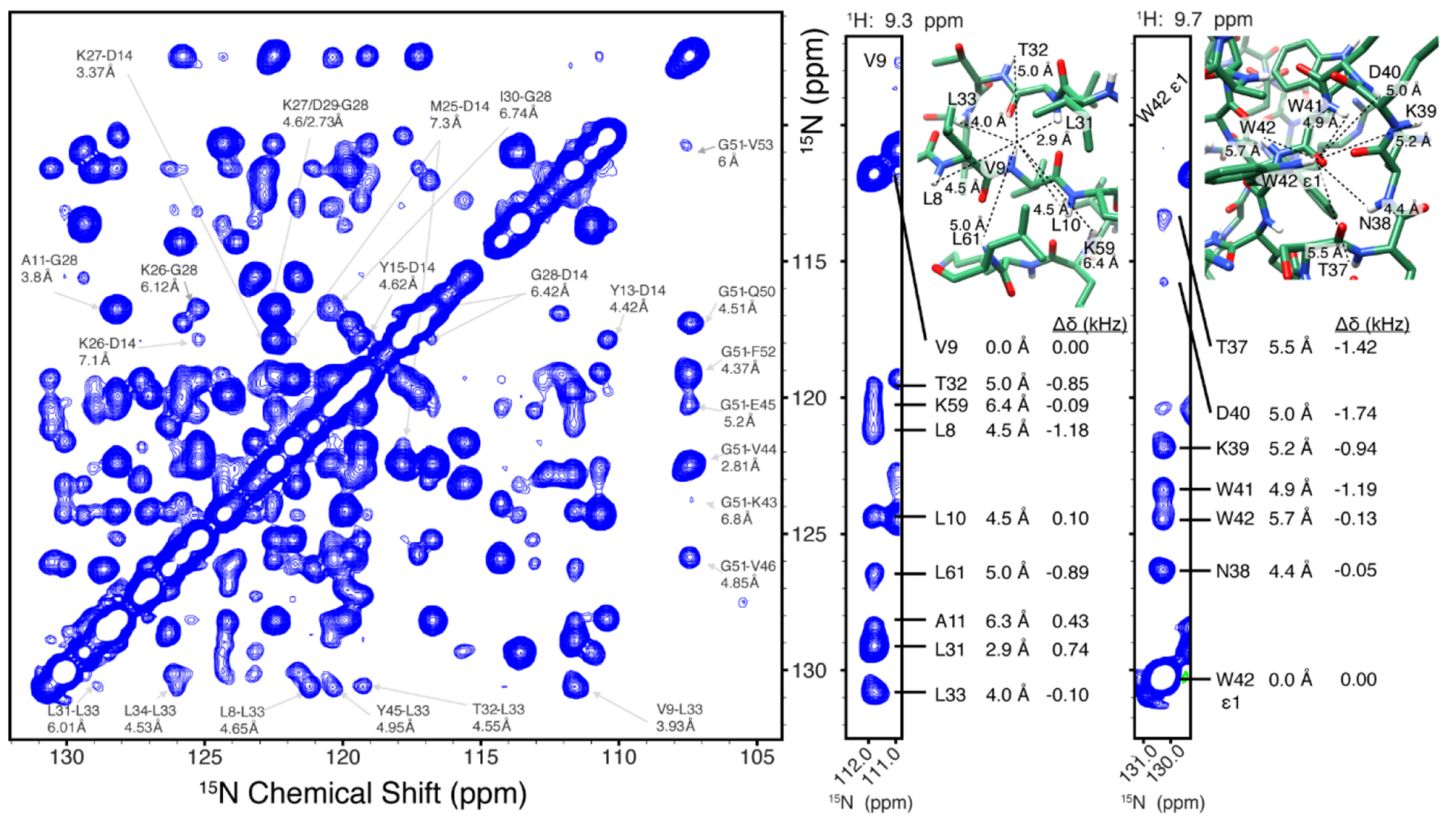
^15^N–^15^N projection of the 3D (H)N(H)(H)NH^MODIST^ spectrum (6.48 ms mixing) recorded at 1200 MHz with 55.555 kHz MAS. Two strips, extracted from the 3D at the proton frequencies of V9 and W42 ε1, are shown at the right, together with assignments for the observed correlations, internuclear distances, and isotropic chemical shift differences (Δδ). Distances were taken from the crystal structure of SH3 (PDB code 2NUZ). The proton carrier frequency was set to 3 ppm for the duration of mixing (further experimental details are given in the Supporting Information).

Because MODIST has minimal dependence on the carrier frequency position, the approach is expected to be suitable for simultaneous Hα–Hα and H_methyl_–H_methyl_ mixing within (H)C(H)(H)CH spectra, where the carrier frequency position is set to −1 ppm. Figure S14 shows the ^13^C–^13^C projection of such a spectrum, recorded on SH3 using a 600 MHz spectrometer. As expected, the peak intensity is reduced far from the diagonal (proton and carbon frequencies of aliphatic moieties are correlated), and Hα–Hα correlations can be observed. Because of the relatively small frequency separation in the aliphatic spectrum, some mixing also occurs between the alpha and methyl regions.

Figure S15 compares MODIST and RFDR for deuterated SH3 using an 850 MHz spectrometer. The frequency selectivity of the method is evident in the suppression of cross-peaks to protons near the extreme edge of the amide region, around 7 ppm. With 30.48 ms MODIST mixing, an additional peak, G51 to L33 (9.63 Å), is detected. This correlation likely arises due to relayed transfer.

We also evaluated MODIST spectra of the uniform ^13^C,^15^N-labeled influenza A M2 membrane protein (Figures 3 and 4). The M2 protein assembles as a dimer of dimers,^58^ such that each residue gives rise to two peaks, here indexed as A and B in Figure 4. Upon comparison of 2D (H)N(H)H^RFDR^ and (H)N(H)H^MODIST^ spectra (Figures S16 and S17), MODIST again shows excellent retention of amide signal, while RFDR efficiently mixes signal into the side-chain.

**Figure 3:**
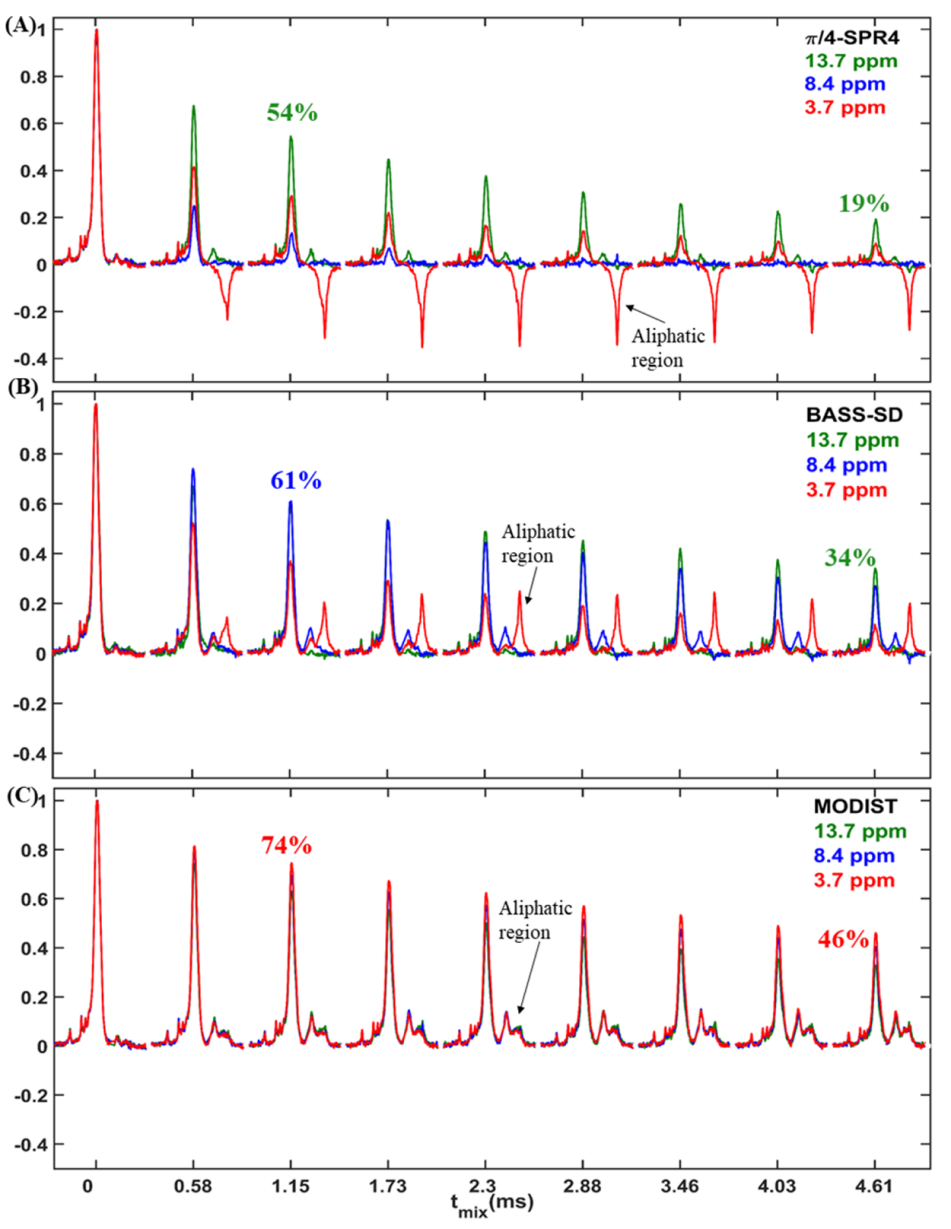
1D (H)N(H)H spectra of the membrane protein influenza A M2 with SPR4_2_ (A), BASS-SD (B), or MODIST (C) mixing. The carrier was set to either 13.7 ppm (green), 8.4 ppm (blue), or 3.7 ppm (red). Descriptions of BASS-SD and SPR pulses are in Figure S21. The artifact peak from water at 4.7 ppm was removed digitally. Data were recorded from a 600 MHz spectrometer with 55.555 kHz MAS.

**Figure 4:**
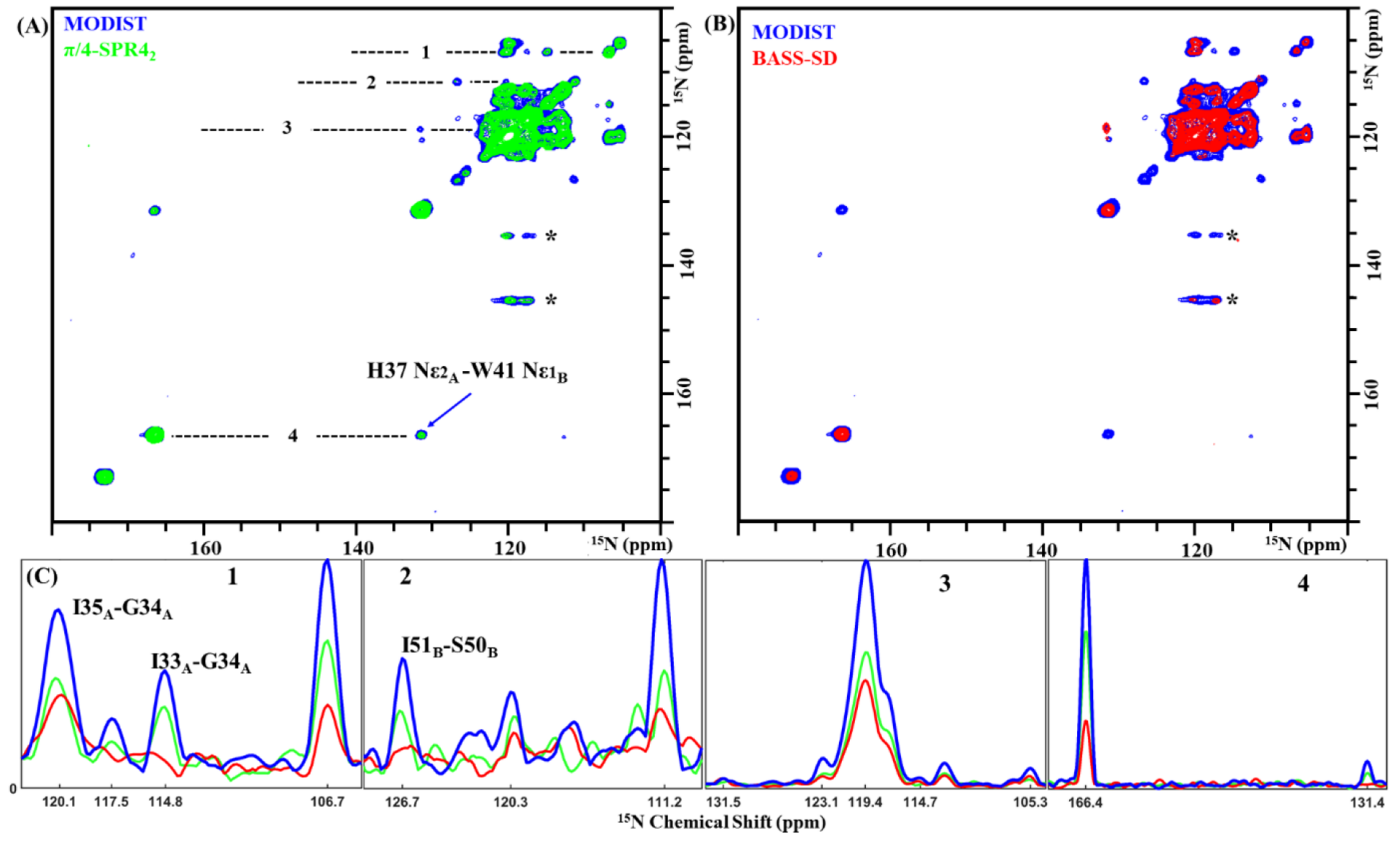
^15^N–^15^N projections of 3D (H)N(H)(H)NH spectra of influenza A M2 comparing MODIST (blue, 2.736 ms mixing, 8.4 ppm carrier frequency) (A) with SPR4_2_ (green, 2.736 ms mixing, 13.7 ppm carrier frequency) and (B) with BASS-SD (green, 4.608 ms mixing, 8.4 ppm carrier frequency). (C) Four slices from the MODIST (blue), SPR4_2_ (green), and BASS-SD (red) spectra, marked with dashed lines in panel A. ∗ indicates artifacts. Data were recorded from a 600 MHz spectrometer with 55.555 kHz MAS. Further experimental details are provided in the Supporting Information.

For M2 at 55.555 kHz, MODIST compares favorably with two selective methods shown at 100 kHz MAS to improve amide–amide transfer with respect to RFDR: BASS-SD^47^ and SPR.^46^Figure 3 compares 1D (H)N(H)H spectra obtained by using BASS-SD, π/4-SPR4_2_, or MODIST, with the proton carrier frequency set to three different values (13.5 ppm, green; 8.4 ppm, blue; 3.7 ppm, red). Of the three, MODIST shows the highest retention of the total amide signal as well as less dependence on the position of the carrier frequency. Table 2 summarizes the normalized intensities of amide signals for all three methods at 4.609 ms mixing. For the three carrier frequencies, the aliphatic region is similar for MODIST, while for BASS-SD and π/4-SPR4_2_ strong aliphatic transfer occurs for certain conditions. Figures S18–S20 show additional 2D (H)N(H)H spectra comparing MODIST, SPR5_4_, and π/4-SPR4_2_. For SH3 at 100 kHz, similar peak intensities were observed for both MODIST and BASS-SD.

**Table 2:** Maximal Intensity (%) of π/4-SPR4_2_, BASS-SD, and MODIST Signals at 4.608 ms Mixing for Three Different Positions of the Carrier Frequency (Intensities Taken from Figure 3)

method	13.7 ppm	8.4 ppm	3.7 ppm
π/4-SPR4_2_	19	0	8
BASS-SD	34	27	11
MODIST	33	40	46

Comparisons of transferred signals are shown in ^15^N–^15^N projections of 3D (H)N(H)(H)NH spectra with MODIST, BASS-SD, or SPR4_2_ mixing (Figure 4). We chose 2.736 ms mixing for comparison of MODIST and SPR4_2_. Typical BASS-SD mixing is longer, and we therefore compare a BASS-SD spectrum at 4.6 ms (a MODIST spectrum at 4.6 ms is shown in Figure S21). Correlations in the (H)N(H)(H)NH^MODIST^ spectrum (blue) have ∼2 times higher intensities than when employing SPR4_2_ (green) and BASS-SD (red), and at least five correlations could only be observed when using MODIST. Cross-peak intensities can be compared in the slices of the ^15^N–^15^N projection shown in Figure 4C. Three additional correlations are observed in the 3D (H)N(H)(H)NH^MODIST^ spectrum with 4.608 ms mixing, two of which were assigned to correlations separated by three residues, which confirms the known helical secondary structure (Figure S21). At 4.6–5 Å, these contacts correspond to relatively long distances. Figure S22 shows the ^15^N–^15^N projection of the corresponding 3D spectrum using SPR5_4_ (1.296 ms mixing), a double quantum mixing sequence.

In summary, we described MODIST, a selective dipolar recoupling sequence, and demonstrated its performance for amide protons in fully protonated samples. MODIST achieves efficient selective transfers for a broad range of carrier frequency values. We presented MODIST spectra of two fully protonated proteins, microcrystalline SH3 and the membrane protein M2, and compared them with the broadband mixing sequence RFDR and two selective methods, BASS-SD and SPR (π/4-SPR4_2_ and SPR5_4_). The advantageous features of MODIST allowed the detection of ^1^H^N^–^1^H^N^ correlations with up to 2-fold improvement in intensity as compared with other state-of-the-art selective dipolar recoupling sequences. The bandwidth of MODIST approximately covers the amide region even at a magnetic field of 28.18 T (a 1200 MHz spectrometer), which is the highest magnetic field currently available for high-resolution NMR.

## Supporting Information Available

Numerical simulations of MODIST, SPR5_4_, π/4-SPR4_2_, and π/2-SPR4_2_; additional experimental data using MODIST, RFDR, BASS-SD, SPR5_4_, and π/4-SPR4_2_ for dipolar recoupling; experimental parameters; Bruker Topspin pulse programs implementing the MODIST sequence (PDF)
