# Supplementary material for: Modest Offset Difference Internuclear Selective Transfer via Homonuclear Dipolar Coupling
Source: J Phys Chem Lett. 2022 Feb 8;13(6):1540–6. doi: 10.1021/acs.jpclett.1c03871 (PMC8859849; doi:10.1021/acs.jpclett.1c03871)
Supplement: Supplementary file 1 — jz1c03871_si_001.pdf [file jz1c03871_si_001.pdf]

Supplementary Information

Modest Offset Difference Internuclear Selective Transfer via Homonuclear Dipolar Coupling

Authors: Evgeny Nimerovsky, Eszter É. Najbauer, Kumar Tekwani Movellan[#], Kai Xue, Stefan Becker & Loren B. Andreas**

Department of NMR based Structural Biology, Max Planck Institute for Biophysical Chemistry, Am Fassberg 11, Göttingen, Germany

[#] Current address: Department of Chemistry and Biochemistry, University of Delaware, Newark, DE 19716, United States.

***Corresponding authors**: land@nmr.mpibpc.mpg.de ORCID: 0000-0003-3216-9065 and evni@nmr.mpibpc.mpg.de ORCID: 0000-0003-3002-0718.

Contents

SIMULATIONS 2
EXPERIMENTAL DATA 16
SH3, 600 MHz, 800 MHz and 1200 MHz 16
Deuterated SH3, 850 MHz 20
M2, 800 MHz 21
M2, 600 MHz 23
EXPERIMENTAL METHODS 27
Simulations 27
Sample Preparation 27
Solid state NMR spectroscopy 28
BRUKER PULSE PROGRAMS 35
2D (H)N(H)H MODIST 36
3D (H)N(H)(H)NH MODIST 38
REFERENCE 41

SIMULATIONS

In this section we give a brief numerical comparison of SPR and MODIST pulses. $SPR5_4$ consists of 20 π/2-pulses with the following phase cycle: 0°, 180°, 144°, 324°, 288°, 108°, 72°, 252°, 216°, 36°, 180°, 0°, 324°, 144°, 108°, 288°, 252°, 72°, 36°, 216°.[1,2] SPR pulses are an excellent tool[2,3] for selectively recoupling spin-spin correlations between different groups (e.g., C_α and C_β). However recoupling spins of the same type of functional groups (for example, $^1H^N$-$^1H^N$) with these pulses is more challenging, since it is difficult to achieve pure double quantum or zero quantum recoupling for all correlations. Therefore, starting with SPR5 we came up with a new sequence, MODIST (Figure S1A), which consists of 16 π/4-pulses with phase cycle: 90°, 270°, 180°, 0°, 180°, 0°, 270°, 90°, 270°, 90°, 0°, 180°, 0°, 180°, 90°, 270°. The length of a single π/4-pulse is $t_p = \frac{T_R}{4}$ (T_R is a rotor period) and rf-field power corresponds to half the MAS rate ($\nu_{rf} = 0.5\nu_R$).

Recently Xiao et al published a study[2] investigating the behavior of SPR5 pulses under different flip angle values, p = π/4, π/2 and 3π4. The difference between MODIST and π/4-SPR4$_2$ sequences is phase cycling. The phase cycling of π/4-SPR4$_2$ is: 90°, 270°, 180°, 0°, 270°, 90°, 0°, 180°, 270°, 90°, 0°, 180°, 90°, 270°, 180°, 0°. Note the different phase starting at the fifth pulse. The difference in the phase cycling changes the properties of SPR pulses, as detailed below.

Figure S1B shows a simulated four spin system – two amide protons (^{N}H) and two aliphatic protons (^{α}H). This spin system was used to obtain Figures S1 and S3-S6. Figures S1C-E show how the offset values of amide protons determine the transferred signal between amide protons (C), the total amide signal (D, the signal of the first amide signal + transferred to the second amide), and the undesired transferred signal from amide proton to the closest aliphatic proton (D). All signals were simulated using 2.016 ms mixing. On the basis of the simulations in Figure S1C, the transferred signal reaches at least 50% of the maximal transfer with respect to the signal with zero offset difference if the offset difference (Δf_{MODIST}) does not exceed ~0.64 kHz. However as shown below in Figures S6-S8, this value can be increased by setting the carrier frequency position to about 3 ppm or even -1 ppm without substantially decreasing the efficiency of MODIST pulses.

Figure S1D displays the high efficiency of MODIST in retaining the initial signal (at least ~98%), whereas Figure S1E shows the low intensity of the undesired transferred signal (at most ~1%). Figure S1F shows the dependence of the MODIST signal on the distances between amide protons, when the offset difference is lower (0 ppm / 0 Hz, solid lines) and larger (1 ppm / 0.8 kHz, dashed lines) than the optimal threshold of 0.7 kHz. In case of 0 offset difference, it is possible to detect long distances, however, for 0.8 kHz offset difference (dashed line) only detection of distances up to about 5 Å can be expected. For any quantification of MODIST signals, the offset differences must be considered.

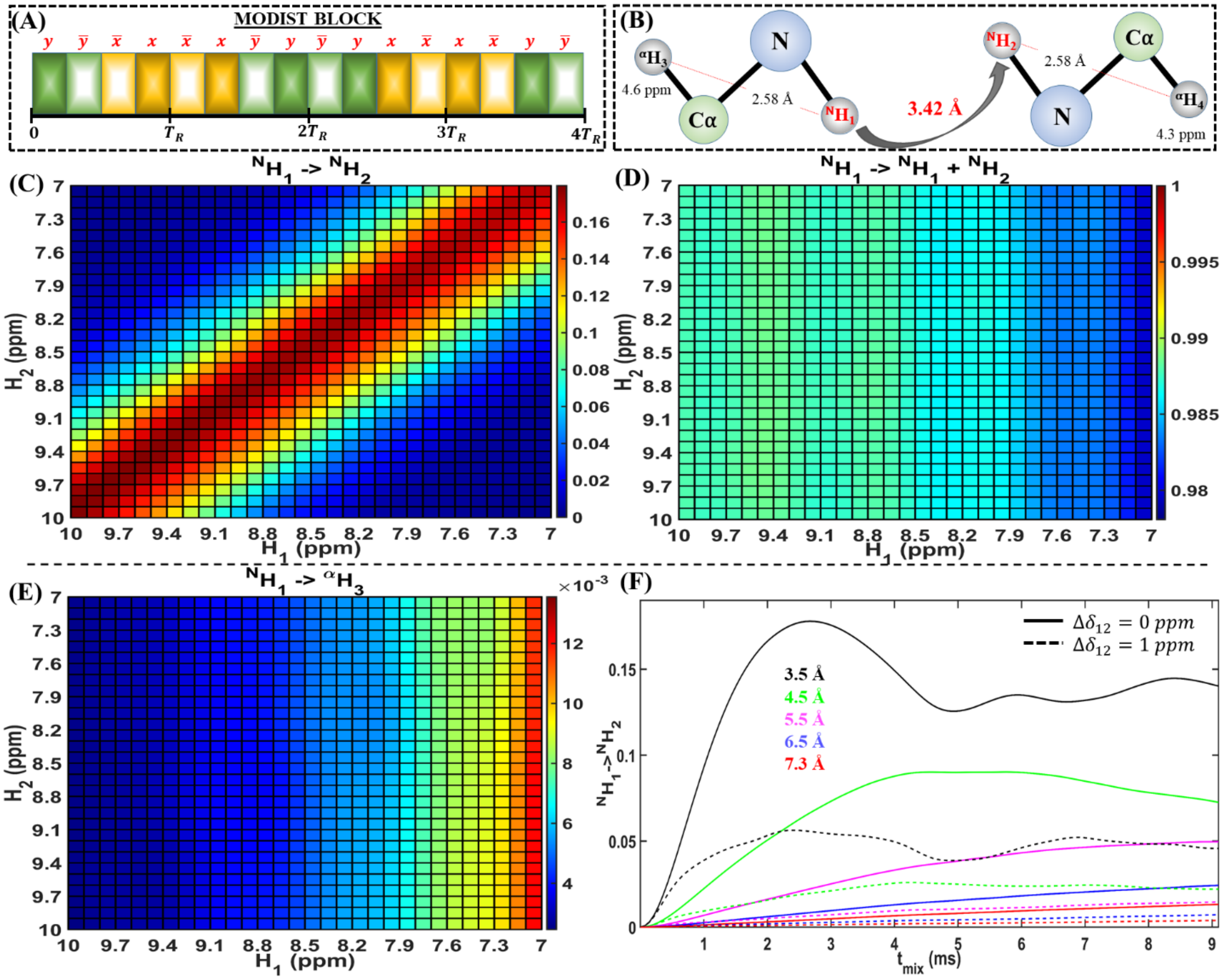

Figure S1 Simulated MODIST mixing for a four spin system. (A) A schematic MODIST block – 16 π/4 pulses with the phase cycle: $y\bar{y}\bar{x}x\bar{x}x\bar{y}y\bar{y}yx\bar{x}x\bar{x}y\bar{y}$. At 55.555 kHz MAS the length of a single pulse is 4.5 μs and the rf-field power is set to 27.777 kHz. (B) Schematic representation of the simulated four spin system. (C)-(E) Simulated amide-amide signal transferred using MODIST as a function of the amide proton offset values. The distance between the amide protons was kept constant (3.42 Å). The carrier frequency was set to 8.2 ppm and the simulated signals were measured at 2.016 ms mixing. (C) The tranferred amide signal. (D) The total amide signal: the signal of the first amide proton + transferred signal to the second amide proton. (E) The undesired transferred amide-aliphatic signal. (F) Simulated MODIST transferred amide-amide signals for various distances: (3.5 Å – black lines; 4.5 Å – green lines; 5.5 Å – pink lines; 6.5 Å – blue lines and 7.3 Å – red lines), and the following offset differences: 0 ppm / 0 Hz (solid lines) and 1 ppm / 0.8 kHz (solid lines). Chemical shift anisotropy (CSA) values were 8 and 7.2 kHz for amide spins

and 6.56 and 6.16 for aliphatic spins. The offset values (in kHz) were simulated using an 800 MHz proton Larmor frequency.

Similar to Figure S1C-E, we simulated the behavior of $SPR5_4$ (Figure S2) and $\pi/4$-$SPR4_2$ (Figure S3-4). For $SPR5_4$ we observe only anti-diagonal transfers as was shown before.[1] Under the same conditions as in Figure S1, $SPR5_4$ transfers a higher fraction of the total signal than MODIST, however, the loss of total signal is very high in this case (up to 28%) (Figure S2B), and the undesired signal (transfer to aliphatic protons) is higher than in the case of MODIST (up to ~16.5% in the absolute value).

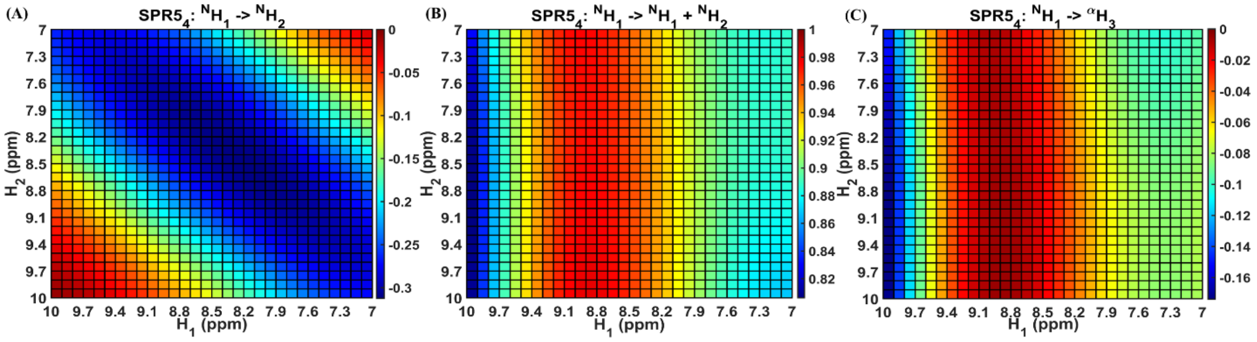

Figure S2 Simulated $SPR5_4$ signals on a four spin system, as a function of amide offset values. Simulated parameters were the same as in Figure S1C-E. (A) Transferred amide-amide signal. (B) The total amide signal: the signal of the first amide proton + transferred signal to a second amide proton. (C) Undesired transferred amide-aliphatic signal. The carrier frequency was set to 8.2 ppm, and the simulated signals were measured at 0.864 ms mixing. The offset values (in kHz) were simulated using 800 MHz proton Larmor frequency.

Decreasing the flip angle of $SPR4_2$ pulses from $\pi/2$ to $\pi/4$ in the simulations, we observe diagonal and anti-diagonal transfers (Figure S3A). It is also shown in Ref. [[2]] for $SPR5_1$ with a flip angle of $\pi/4$. For p-$SPR4_2$ pulses (p indentifies the flip angle[2]) with p= $\pi/2$ and $\pi/4$ the transfer efficiency is worse than that of other SPR pulses. The antigonal transfers achieve ~17% (in absolute value) in the best case with $\pi/2$, while for $SPR5_4$ it achieves 60% in the absolute value

(Figure S2A). The loss of the total signal is especially high around zero offset (~8.2 ppm, Figure S3B), however not as so high as for SPR5_4. With respect to SPR5_4, π/4-SPR4_2 shows less undesired transfer to aliphatic protons (Figures S2C and S3C).

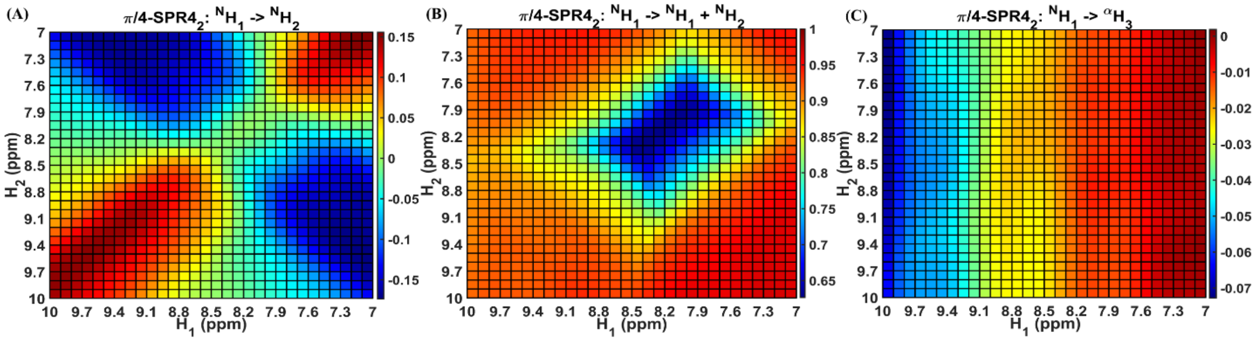

Figure S3 π/4-SPR4_2 signals simulated on a four spin system, as a function of amide offset values setting the carrier frequency to 8.2 ppm. Simulated parameters were the same as in Figure S1C-E. (A) Transferred amide-amide signal. (B) The total amide signal: the signal of the first amide proton + transferred signal to a second amide proton. (C) Undesired amide-aliphatic signal transfer. The simulated signals were measured at 2.016 ms mixing. The offset values (in kHz) were simulated using a proton Larmor frequency of 800 MHz proton.

One of our solutions for increasing the efficiency of π/4-SPR4_2 is setting the carrier frequency (CF) away from the amide and aliphatic range, e.g., at 13.5 ppm. Although the supression of the undesired transfers as good as for MODIST (Figure S4C), the weak antidiagonal (Figure S4A) is still observed. Under these conditions π/4-SPR4_2 pulses have 33% smaler Δf than MODIST pulses. Additionally, the tranfer efficiency of π/4-SPR4_2 pulses depends on the offset values of the spins with respect to position of CF. For example, around 8.2 ppm the transferred signals are ~1.15 fold lower than around 9.5 ppm region (Figure S4A). This makes π/4-SPR4_2 less efficient than MODIST for the amide-amide transfer.

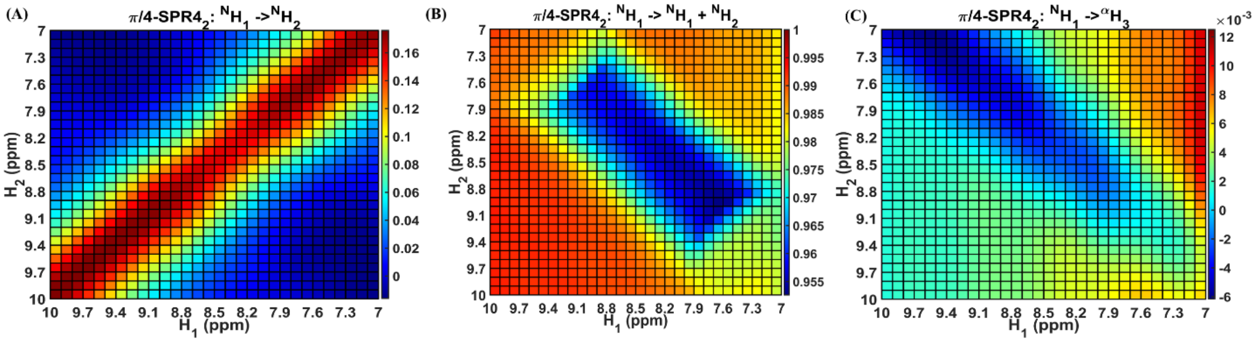

Figure S4 Simulated π/4-SPR4$_2$ signals in a four spin system as a function of amide offset values setting the carrier frequency to 13.5 ppm. Simulated parameters were the same as in Figure S1C-E. (A) Transferred amide-amide signal. (B) The total amide signal: the signal of the first amide proton + transferred signal to a second amide proton. (C) The undesired transferred amide-aliphatic signal. The simulated signals were measured at 2.016 ms mixing. The offset values (in kHz) were simulated using 800 MHz proton Larmor frequency.

Figure S5 shows simulated (A, B, D, E) and experimental (C) MODIST signals, demonstrating how flip angle values influence the MODIST tranfers.

Increasing the flip angle of MODIST pulses from π/4 to π/2, Δf_{MODIST} increases from 0.64 kHz to 1.7 kHz (Figure S5A). However, the maximal transfer efficiency is decrease (~1.4 fold) and the total amide signal retains less (Figure S5B). The comparison of 2D SH3 (H)N(H)H^{MODIST} spectra with π/4 (blue) and π/2 (red) flip angles (Figure S5C) confirms the loss of efficiency seen in simulations. With flip angles of π/2 (red) the amide region appears with significantly lower intensities than with π/4 (blue) (it is clearly seen in slice I in Figure S5C). Additionaly, undesired amide-aliphatic transfers are also observed in the first case.

Figure S5D-E demonstrates the MODIST simulations in which the flip angle was set to π/8. Δf_{MODIST} is significantly smaller in this case and is 0.24 kHz.

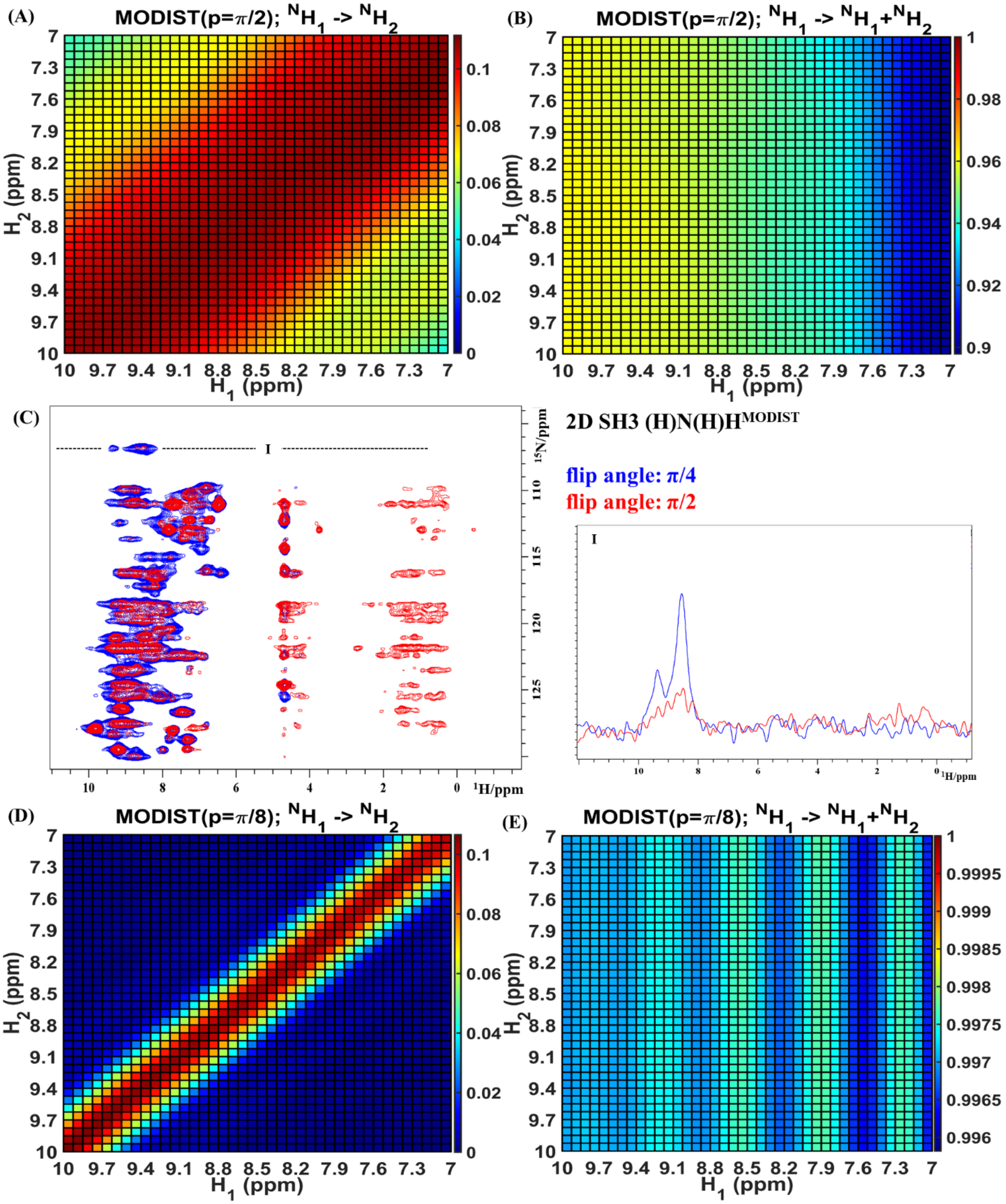

Figure S5 (A, B, D, E) MODIST signals simulated on a four spin system, as a function of amide offset values and flip angles: (A), (B) : π/2; (D), (E) : π/8. Simulated parameters were the same as in Figure S1C-E, except CSA values, which were 1 and 4 kHz for amide and 2 and 3 kHz for aliphatic spins. Offset values (in kHz) were simulated using a

proton Larmor frequency of 800 MHz. (C) 2D (H)N(H)H^{MODIST} SH3 experiments acquired at an 800 MHz spectrometer with π/4 (1.152 ms mixing) and π/2 (1.008 ms mixing). I: A slice from 2D MODIST experiments, flip angle: π/4 (blue); π/2 (red). Experimental details of all experiments are provided in the experimental methods, below.

The MODIST simulations in Figure S1 were obtained setting the CF position to 8.2 ppm. Figure S6 demonstrates the influence of the amide proton spin offsets on the amide-amide transfer (A, C, E, G) and on the total amide signal (B, D, F, H) under four different settings of CF. Although MODIST's performance is less sensitive to the CF than that of other selective methods, its effect on the Δf_{MODIST} is not negligible. Table S1 summarizes the Δf_{MODIST} values.

CF(ppm)	12.7	4.6	1.6	-1
Δf_{MODIST} (kHz)	0.6	0.84	0.96	1.15

Table S1 Maximal offset difference values with which the transferred signal is at least 50% of the maximal transfer (Δf_{MODIST}) under different settings of carrier frequency (CF).

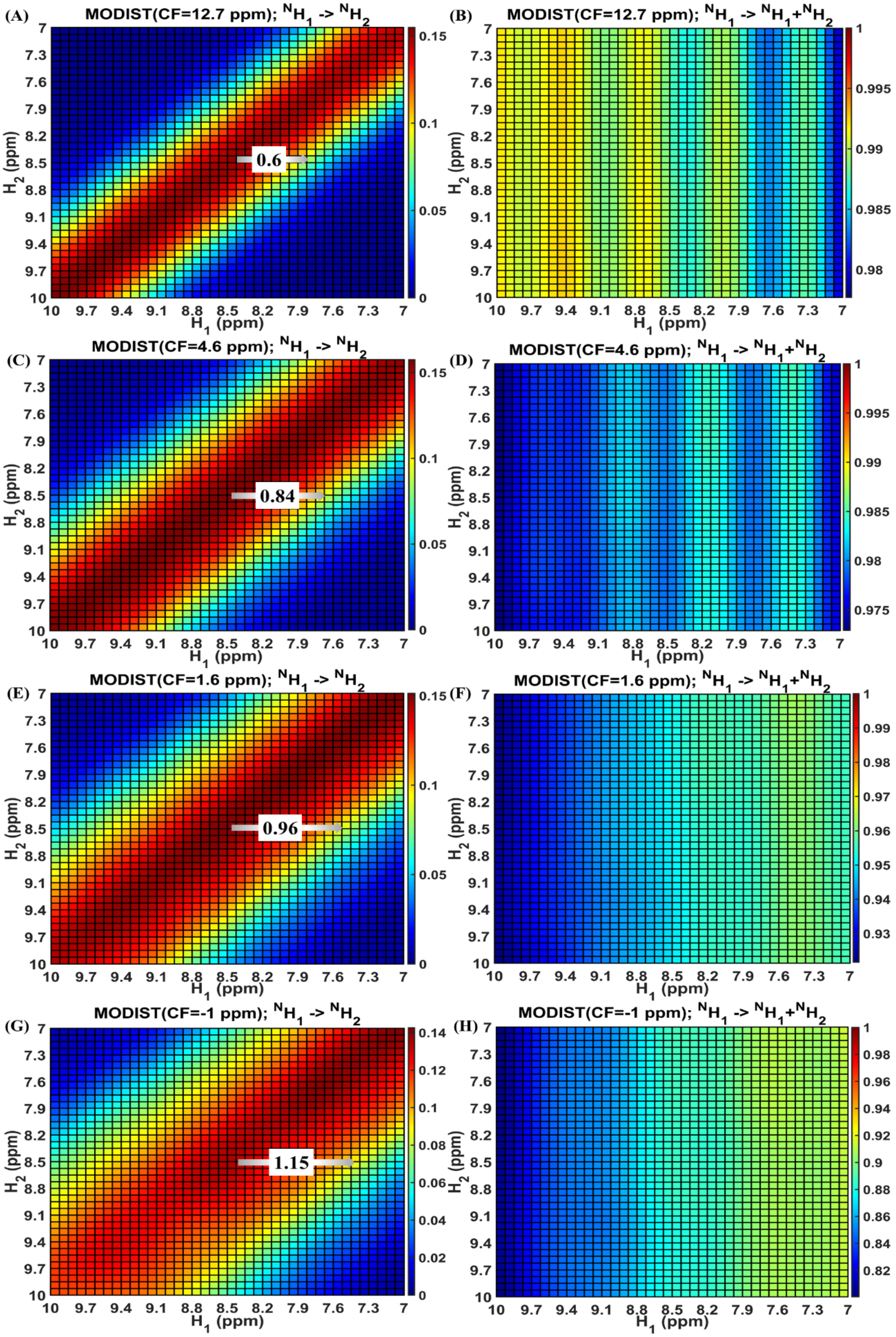

(A)
MODIST(CF=12.7 ppm); $^{N}H_1$ -> $^{N}H_2$
0.6
(B)
MODIST(CF=12.7 ppm); $^{N}H_1$ -> $^{N}H_1$+$^{N}H_2$
(C)
MODIST(CF=4.6 ppm); $^{N}H_1$ -> $^{N}H_2$
0.84
(D)
MODIST(CF=4.6 ppm); $^{N}H_1$ -> $^{N}H_1$+$^{N}H_2$
(E)
MODIST(CF=1.6 ppm); $^{N}H_1$ -> $^{N}H_2$
0.96
(F)
MODIST(CF=1.6 ppm); $^{N}H_1$ -> $^{N}H_1$+$^{N}H_2$
(G)
MODIST(CF=-1 ppm); $^{N}H_1$ -> $^{N}H_2$
1.15
(H)
MODIST(CF=-1 ppm); $^{N}H_1$ -> $^{N}H_1$+$^{N}H_2$
H_1 (ppm)
H_2 (ppm)

Figure S6 Simulated MODIST signals in a four spin system as a function of amide offset values. Four different carrier frequencies (CF) were used : 12.7 ppm – (A), (B); 4.6 – (C), (D), 1.6 ppm – (D), (F) and -1 ppm – (G), (H). Simulated parameters were the same as in Figure S1C-E, except CSA values, which were 1 and 4 kHz for amide and 2 and 3 kHz for aliphatic spins. (A), (C), (E), (G) Transferred amide-amide signal. The arrow with values (in kHz) indicate the maximal offset difference values with which the transferred signal is at least 50% of the maximal transfer. (B), (D), (F), (H) The total amide signal (signal of the first amide proton + transferred signal to a second amide proton). Offset values (in kHz) were simulated using 800 MHz proton Larmor frequency.

Δf_{MODIST} has a minimal value of 0.6 kHz setting a CF at 12.7 ppm (Figure S6A), and reaches the largest value at a CF of -1 ppm (1.15 kHz) (Figure S6G). However, with setting CF to -1 ppm the amide protons obtain larger offset values than in other settings. In that case, for some regions MODIST pulses become less efficient. For example around 10 ppm the transferred signals are ~1.4 times lower (Figure S6G) and the loss of the signal is higher (Figure S6H) than for the region around 8 ppm (Figure S6G). It is interesting that experimentally we observed higher retention of the total amide signal with -1 ppm (Figure S7B, red) of CF setting than with 12.7 ppm (Figure S7A, red). Also the undesired amide-aliphatic correlations are observed with 12.7 ppm CF setting (Figure S7A, red).

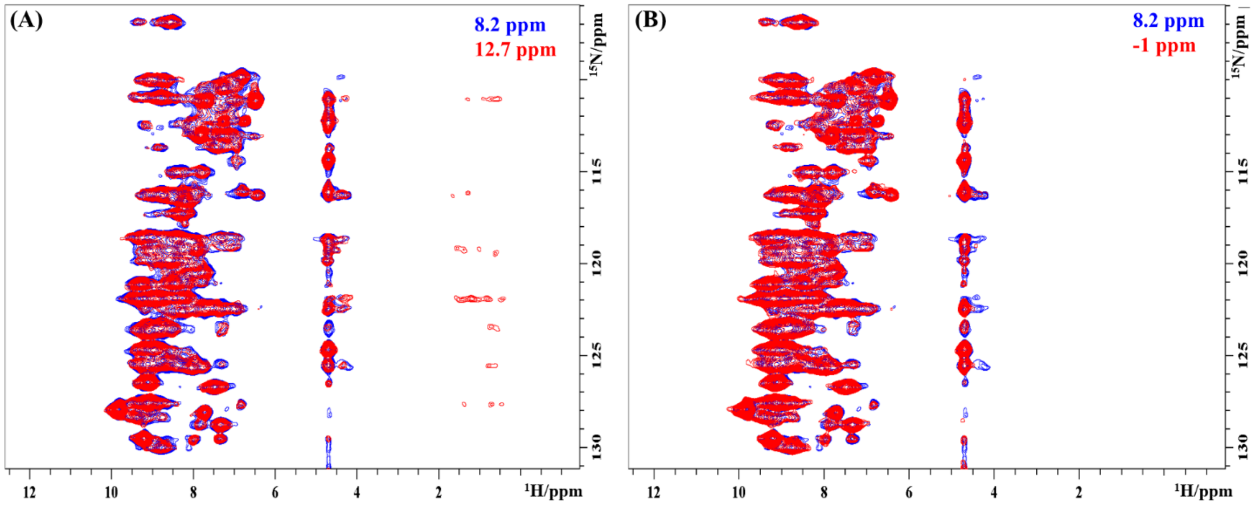

Figure S7 2D (H)N(H)H^{MODIST} spectra of of fully protonated SH3 acquired at an 800 MHz spectrometer using 2.016 ms mixing. The proton CF was set to 12.7 ppm (A, red), -1 ppm (B, red), and 8.2 ppm (blue in both A and B). Experimental details of all experiments are provided in the experimental methods, below.

The phase cycling of MODIST was selected such that the transfer between amide spins and retention the total amide signal are both maximized, while the transfers between amide and aliphatic spins are minimized. The phases of the MODIST pulse sequence are, 90°, 270°, 180°, 0°, 180°, 0°, 270°, 90°, 270°, 90°, 0°, 180°, 0°, 180°, 90°, 270°. Note that the last 8 pulses are constructed with a 180 degree phase shift. A particular set of phases following this rule, and the jump return rule can then be written as QQ' (Q' pulses shifted 180 degrees). In shorthand, listing only the jump pulses of Q (every other pulse) the phases are [90°, 180°, 180°, 270°]. In general, since the phase of the first pulse is arbitrary, we set it to 90°, and there are then 64 different phase sequences. Figure S8A shows the simulated transferred signal between amide spins as a function of the offset difference for all 64 phase sequences. Some phase sequences have a similar efficiency as MODIST (Figure S8A, red line).

Figures S8B-D compare the experimental 1D (H)N(H)H SH3 spectra obtained with the most promising 8 different phase sequences for different positions of the carrier frequency. In general, MODIST has less dependence on CF position. With 8.4 ppm CF position the phase sequences [90°, 0°, 0°, 270°] (B, blue) and [90°, 90°, 0°, 0°] (B, green) show a little higher retention of the total amide signal than MODIST (B, red). However, for other CF positions (C and D) MODIST has higher signal, especially for the -1 ppm case (D).

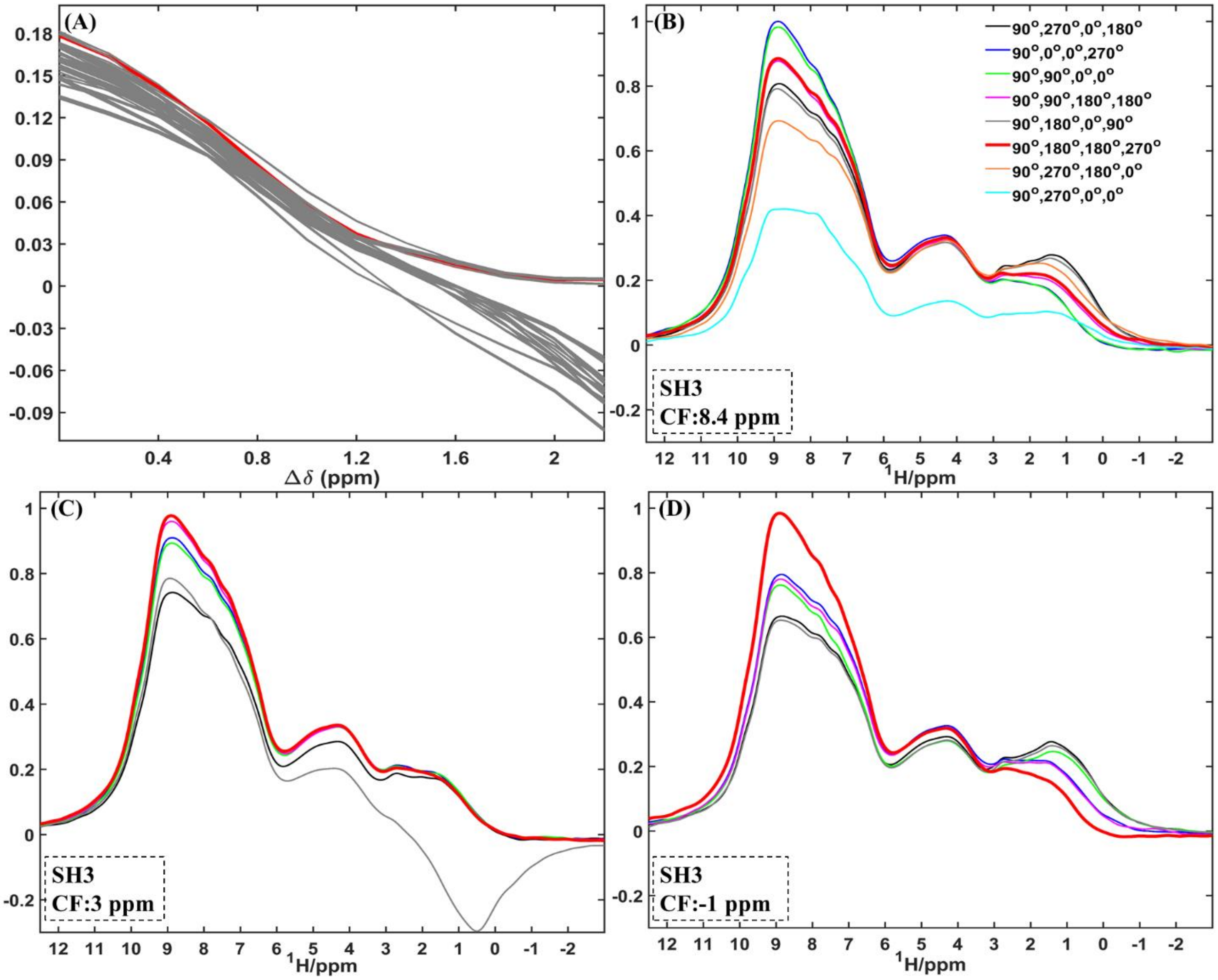

Figure S8 Evaluation of different phase cycles during transfer with 45 degree jump-return pulses (A) Simulated evaluation of the efficiency of the 64 different phase cycles as a function of the offset difference between amide spins. MODIST transferred signal is shown in red. Simulated parameters were the same as in Figure S1C-E, except the CF position, which was 8.4 ppm. (B)-(D) 1D (H)N(H)H SH3 spectra (6.48 ms mixing time) acquired at an 600 MHz spectrometer with different phase cycles and different carrier frequency (CF) positions: (B) – 8.4 ppm; (C) – 3 ppm and (D) – -1 ppm. For (B) 8 phase cycles were investigated: 90°, 270°, 0°, 180° – black; 90°, 0°, 0°, 270° – blue; 90°, 90°, 0°, 0° – green; 90°, 90°, 180°, 180° – pink; 90°, 180°, 0°, 90° – gray; 90°, 180°, 180°, 270° – red (MODIST); 90°, 270°, 180°, 0° – orange and 90°, 270°, 0°, 0° – cyan. (The above phase cycles are for the first pulse of a jump-return element, where the phase of the second pulse of the jump-return is shifted by 180 degrees). For (C-D) only the first six phase cycling schems were evaluated.

Figure S9 compares the simulated signal as a function of offset difference, Δδ, at three different MAS rates (A), with and without CSA values at different external magnetic fields (B) and as a function of the distance between amide spins (C-D).

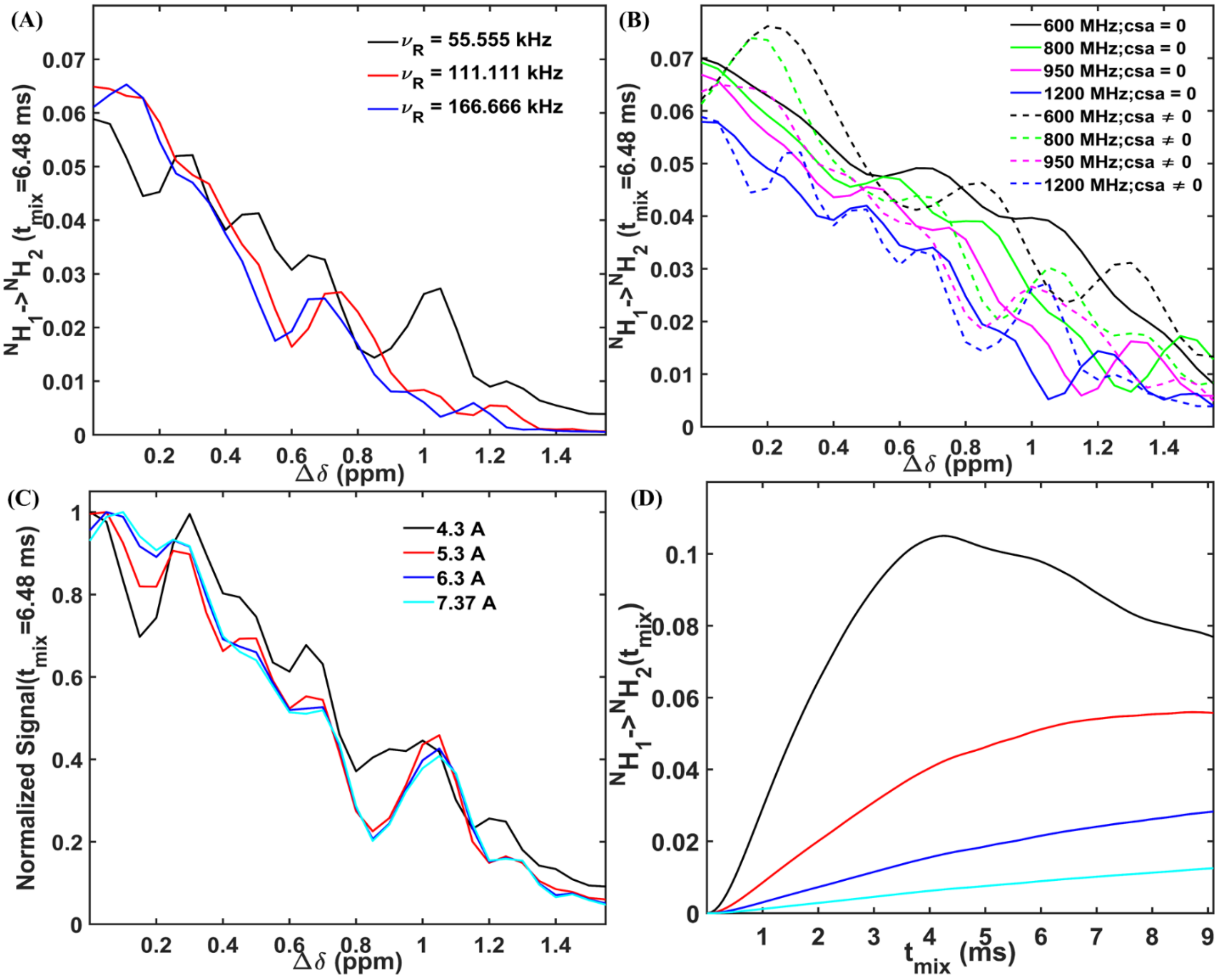

Figure S9 (A-C) Simulated MODIST transferred signal (at 6.48 ms mixing time) as a function of the offset difference between amide spins. A four-spin system was simulated (two amide and two aliphatic spins), where the isotropic chemical shift of the first amide spin was 10 ppm and the isotropic chemical shift of the second was changed from 10 to 8.45 ppm. (A) MAS rate is a variable: 55.555 kHz – black; 111.111 kHz – red and 166.666 kHz – blue. The distance between amide spins was 5 Å. The offset and CSA values [10;9;8.2;7.7] (in ppm) were calculated with respect to the 1200 MHz proton Larmor frequency. (B) The proton Larmor frequency is a variable: 600 MHz – black; 800 MHz – green; 950 MHz – pink and 1200 MHz – blue. Solid lines indicate zero CSA values,

while dashed lines indicate the presence CSA values – 10 and 9 ppm for the amide spins and 8.2 and 7.7 for aliphatic spins. 55.555 kHz MAS was used. The distance between amide spins was 5 Å. (C) The distance between amide spins is a variable: 4.3 Å – black; 5.3 Å – red; 6.3 Å – blue and 7.37 Å – cyan. The offset and CSA values [10;9;8.2;7.7] (in ppm) were calculated with respect to 1200 MHz proton Larmor frequency. 55.555 kHz MAS was used. The signal was normalized to the highest intensity. (D) The simulated MODIST signal as a function of the mixing time under different amide-amide distances (the simulated parameters are the same as in C). In all simulations, CF was set to 3 ppm.

Figure S10 shows the experimental performance of MODIST (6.48 ms mixing) at 100 kHz MAS for fully protonated SH3 microcrystals. The spectrum in Figure S10A shows qualitatively similar transfer characteristics, and confirms the simulations. At this mixing time, amid-amide transfer is observable, and signal starts to build up in the alpha region of the aliphatic protons. The inset shows a comparison of the 1D signal at 0 and 6.48 ms mixing. High preservation of the total amide signal of 80% is observed.

Figure S10B shows two 2D (H)N(H)H^{MODIST} (blue) and (H)N(H)H$^{BASS\text{-}SD}$ (red) spectra. We observe similar transfer efficiency with both methods for this sample.

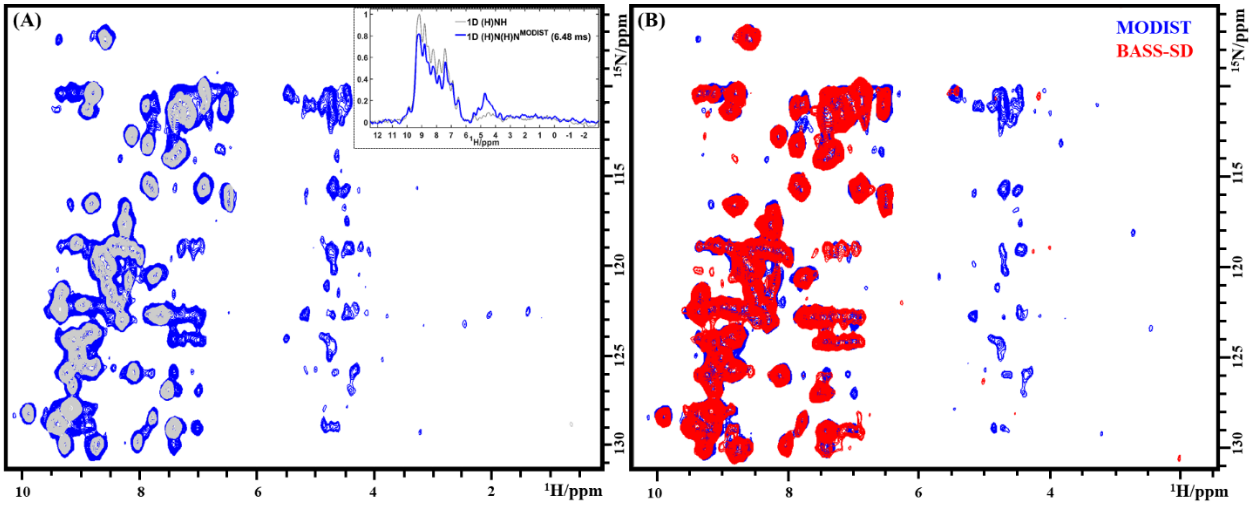

Figure S10 2D spectra of fully protonated SH3 acquired at an 950 MHz spectrometer with 100 kHz MAS. (A) 2D (H)N(H)H^{MODIST} (blue, 6.48 ms mixing time) and (H)NH (gray). The proton CF was set to 3 ppm. (B) 2D (H)N(H)H^{MODIST} (blue, 3 ppm proton CF) and 2D (H)N(H)H$^{BASS\text{-}SD}$ (red, 8.4 ppm proton CF) with 6.48 ms mixing time for both cases. Experimental details are provided in the experimental methods, below.

The above demonstrates with simulations and with confirmation via experiments that the efficiency of MODIST pulses have a small dependence on the CF position. A proton CF can be set around 3 ppm, which will allow to obtain about 0.9 kHz bandwidth (Δf_{MODIST}) and at the same time retain a high level of overall signal.

EXPERIMENTAL DATA

SH3, 600 MHz, 800 MHz and 1200 MHz

Figure S11 shows 2D (H)N(H)H^{MODIST} (blue), (H)N(H)H^{RFDR} (red, $XY4^1_4$ phase cycling[4]) spectra under 4 different mixing times. The data was recorded at a 600 MHz spectrometer.

Figure S12 shows 2D (H)N(H)H^{MODIST} (blue), (H)N(H)H^{RFDR} (red) spectra under 8 different mixing times. The 2D (H)NH reference experiment is shown in gray. The data was recorded at an 800 MHz spectrometer.

Figure S13 shows the ^{15}N-^{15}N projection of the 3D (H)N(H)(H)NH^{MODIST} experiment with 2.16 ms (A, 800 MHz) and 6.48 ms (B, 1200 MHz) mixing with assignment based on the known chemical shifts and the SH3 structure.[5–7]

Figure S14 shows the ^{13}C-^{13}C projection of the 3D (H)C(H)(H)CH ^{MODIST} spectrum with 5.04 ms (A). The data was recorded at a 600 MHz spectrometer. The assignments of the peaks were taken from previous reports.[8,9]

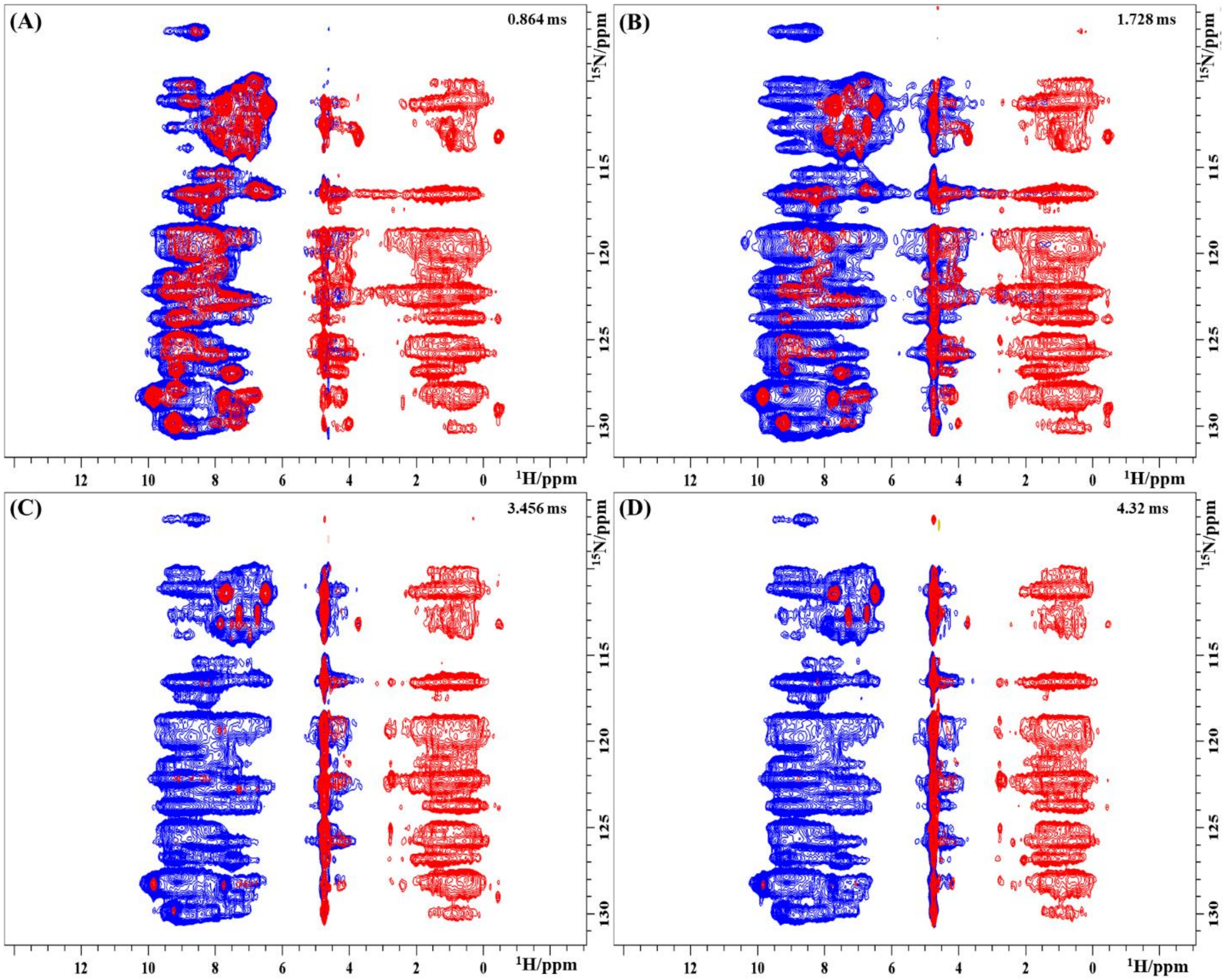

Figure S11 2D (H)N(H)H SH3 spectra as a function of the mixing time (in ms): (A) 0.864, (B) 1.728, (C) 3.456 and (D) 4.32 ms. Spectra acquired with MODIST mixing are shown in blue, with RFDR mixing (with $XY4_4^1$ phase cycling) shown in red. For RFDR and MODIST, the proton carrier frequency was set to 8.4 and 3 ppm, respectively. 55.555 kHz MAS was used. The experimental details of all experiments are provided in the experimental methods, below.

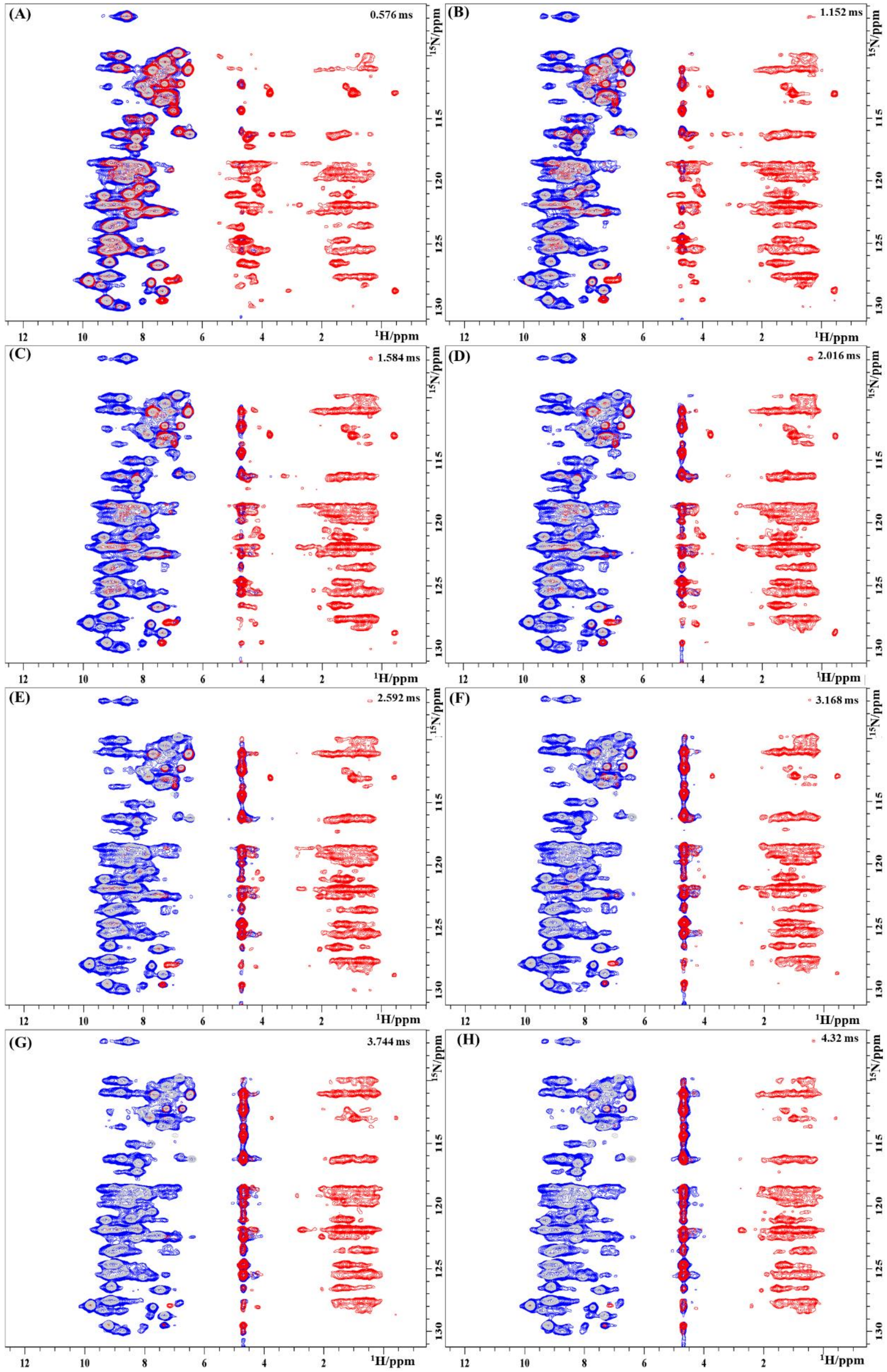
(A)
0.576 ms
(B)
1.152 ms
(C)
1.584 ms
(D)
2.016 ms
(E)
2.592 ms
(F)
3.168 ms
(G)
3.744 ms
(H)
4.32 ms
^{15}N/ppm
^{1}H/ppm
115
120
125
130
12
10
8
6
4
2

Figure S12 2D (H)N(H)H SH3 spectra as a function of the mixing time (in ms): (A) 0.576, (B) 1.152, (C) 1.584, (D) 2.016, (E) 2.592, (F) 3.168, (G) 3.744 and (H) 4.32 ms. Spectra acquired with MODIST mixing are shown in blue, with RFDR mixing are shown in red, and the the (H)NH reference spectrum is shown in gray. For all experiments the proton carrier frequency was set to 8.2 ppm and 55.555 kHz MAS was used. The experimental details of all experiments are provided in the experimental methods, below.

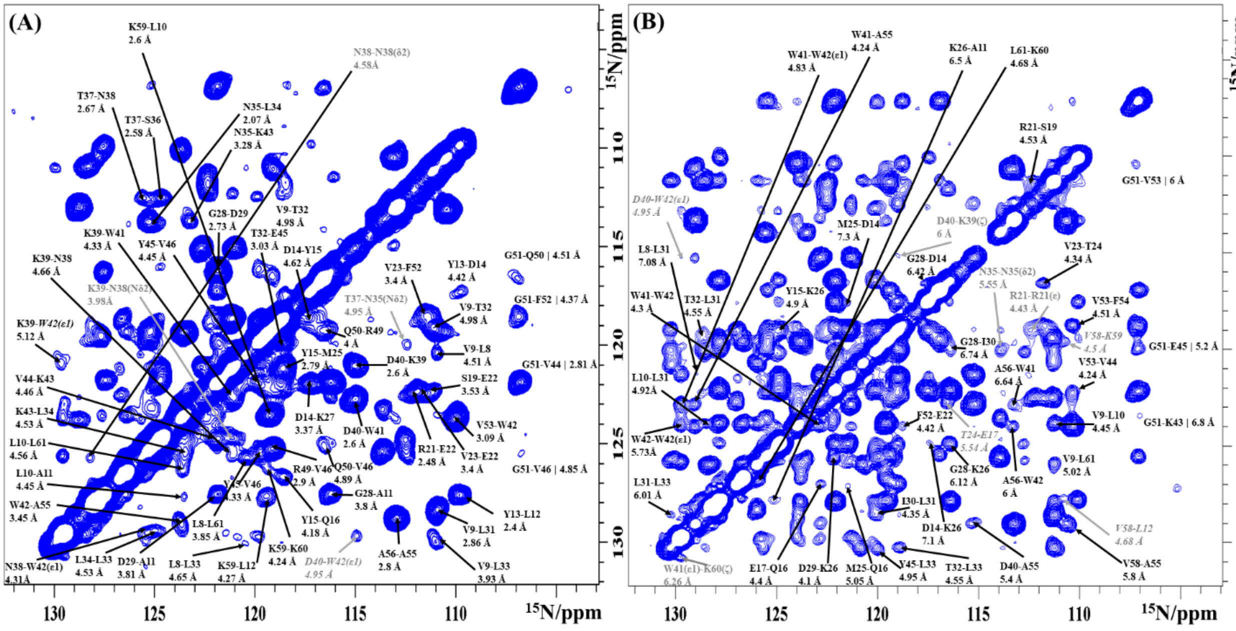

Figure S13 ^{15}N – ^{15}N projection of the 3D (H)N(H)(H)NH^{MODIST} spectrum. For peaks with black labels, both ^{15}N isotropic chemical shifts could be assigned; for peaks labeled in gray, only one ^{15}N assignment was known or the symmetric cross-peak above the diagonal is not well resolved or not observed. In these cases, the unkown resonance was assigned to the closest unasigned ^{15}N nucleus, including side chain resonances (based on the crystal structure, PDB: 2NUZ). In Figure B only new peaks are assigned. (A) 800 MHz, 2.016 ms mixing. The proton carrier frequency was set to 8.2 ppm for the duration of mixing. (B) 1200 MHz, 6.48 ms mixing. The proton carrier frequency was set to 3 ppm for the duration of mixing. For both spectra, 55.555 kHz MAS was used. The experimental details of all experiments are provided in the experimental methods, below.

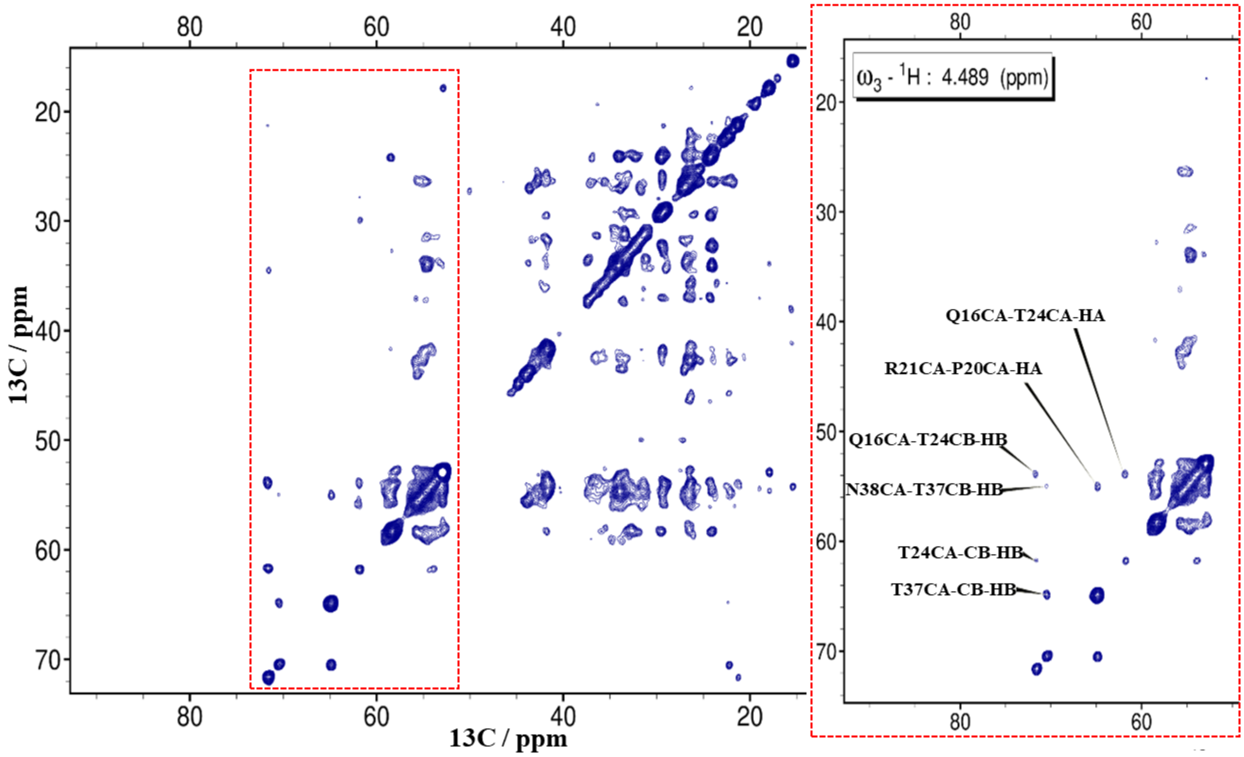

Figure S14 ^{13}C-^{13}C projection of the 3D (H)C(H)(H)CH^{MODIST} spectrum with 5.04 ms mixing. The proton carrier frequency was set to -1 ppm. 55.555 kHz MAS was used. In the red frame, the H$^{\alpha}$/H$^{\beta}$ proton chemical shifts of T24 residue are selected. The experimental details of all experiments are provided in the experimental methods, below.

Deuterated SH3, 850 MHz

Figure S15A shows 2D (H)N(H)H^{MODIST} (blue, 30.48 ms mixing), (H)N(H)H^{RFDR} (red, 9.504 ms mixing) spectra. The mixing times of both spectra were selected by finding when ~50% loss of the total amide signal was observed.

Figure S15B shows the ^{15}N-^{15}N projection of the (H)N(H)(H)NH^{MODIST} spectrum with 30.48 ms mixing.

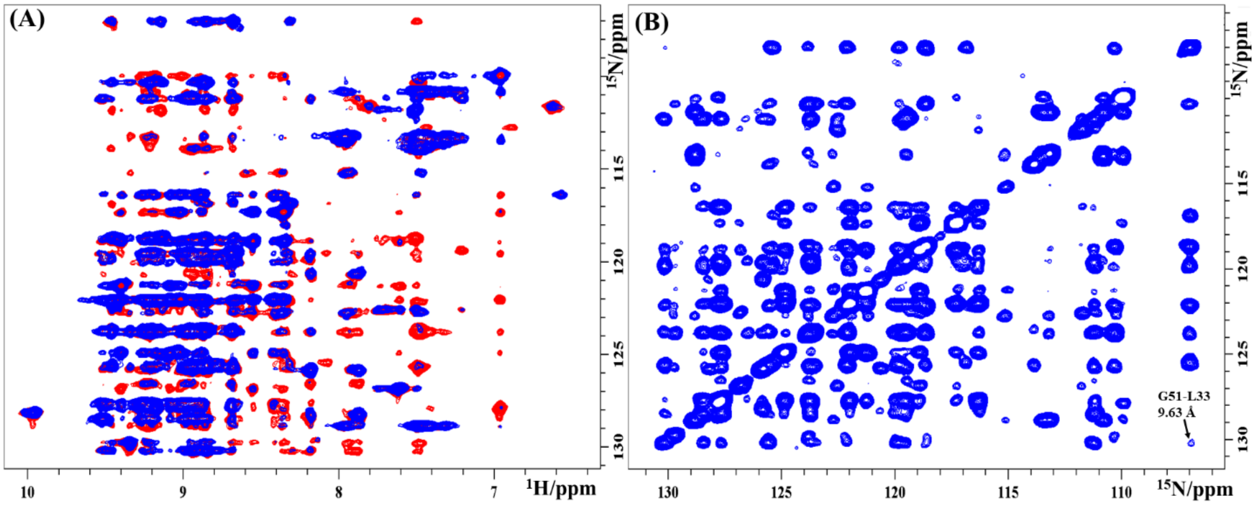

Figure S15 MODIST and RFDR spectra of perdeuterated SH3. (A) 2D (H)N(H)H RFDR (red, 9.504 ms mixing) and MODIST (blue, 30.48 ms mixing) spectra. In each case, the mixing results in 50% loss of total amide signal. For RFDR and MODIST mixing the proton carrier frequency was set to 8.4 and 3 ppm, respectively. (B) Shows the ^{15}N-^{15}N projection of the 3D (H)N(H)(H)NH^{MODIST} spectrum. 55.555 kHz MAS was used. The experimental details of all experiments are provided in the experimental methods, below.

M2, 800 MHz

Figure S16A is a comparison of 1D (H)N(H)H spectra acquired with MODIST (blue) and RFDR (red) recoupling. Figure S16B shows 2D (H)N(H)H^{MODIST} and (H)N(H)H^{RFDR} spectra acquired with 1.296 ms mixing. The build-up of selected cross peaks as a function of mixing time is shown in Figure S16C.

Figure S17 shows 2D (H)N(H)H^{MODIST} (blue), (H)N(H)H^{RFDR} (red) spectra at 4 different mixing times. The 2D (H)NH reference spectrum is shown in grey.

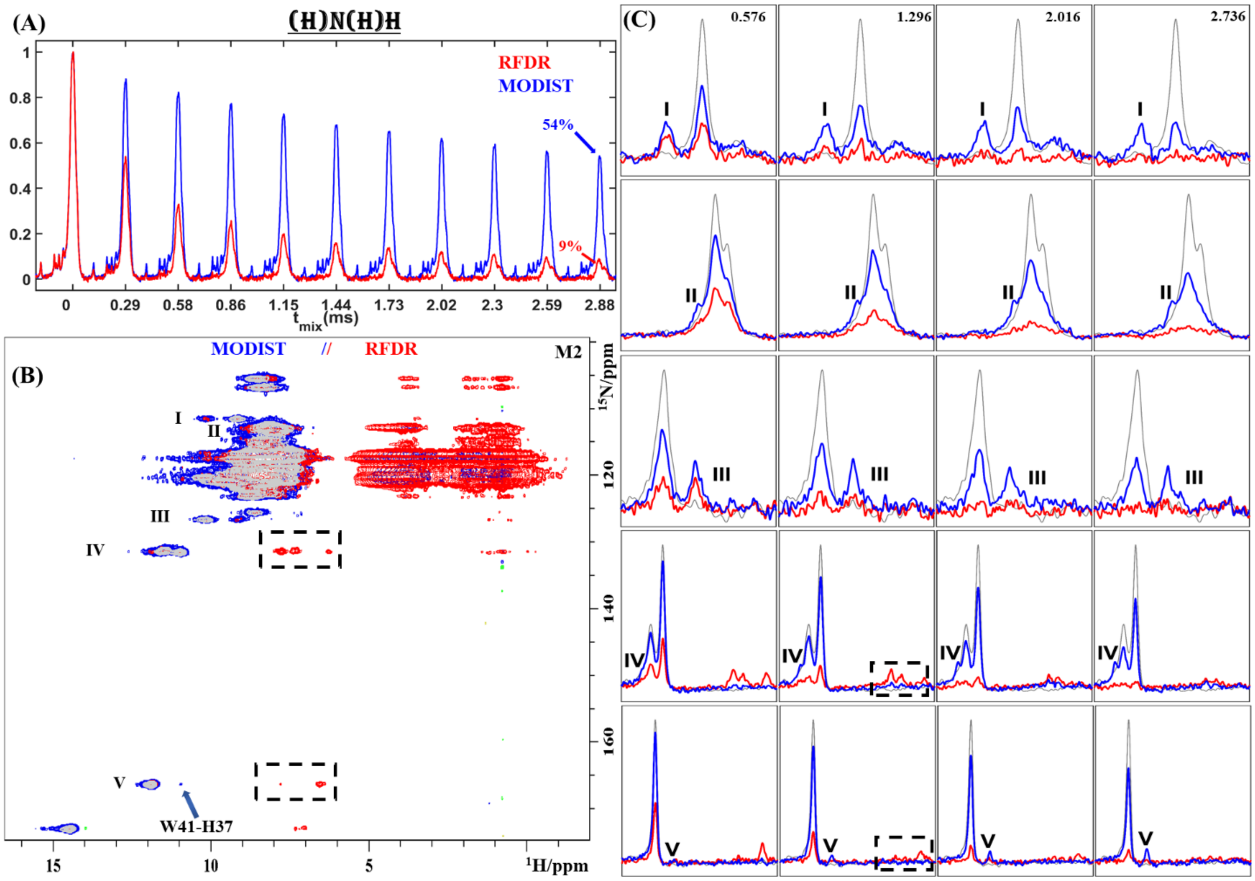

Figure S16 (A) 1D (H)N(H)H spectra of influenza A M2 using MODIST (blue) and RFDR (red) for dipolar recoupling. (B) (H)N(H)H^{MODIST} (blue) and (H)N(H)H^{RFDR} (red) spectra acquired with 1.152 ms mixing. The (H)NH reference spectrum is shown in grey. Dashed rectangles show the intraresidue correlations between amide and aromatic protons, seen with only RFDR. (C) Five slices from (H)N(H)H^{RFDR} (red) and (H)N(H)H^{MODIST} (blue) spectra as a function of 4 mixing times (in ms): 0.576, 1.296, 2.016, and 2.736. The slices are indicated with roman numbers. For all spectra, the proton carrier frequency was set to 8.2 ppm and 55.555 kHz MAS was used. An 800 MHz spectrometer was used. Slices from the reference (H)NH experiment are shown in grey.

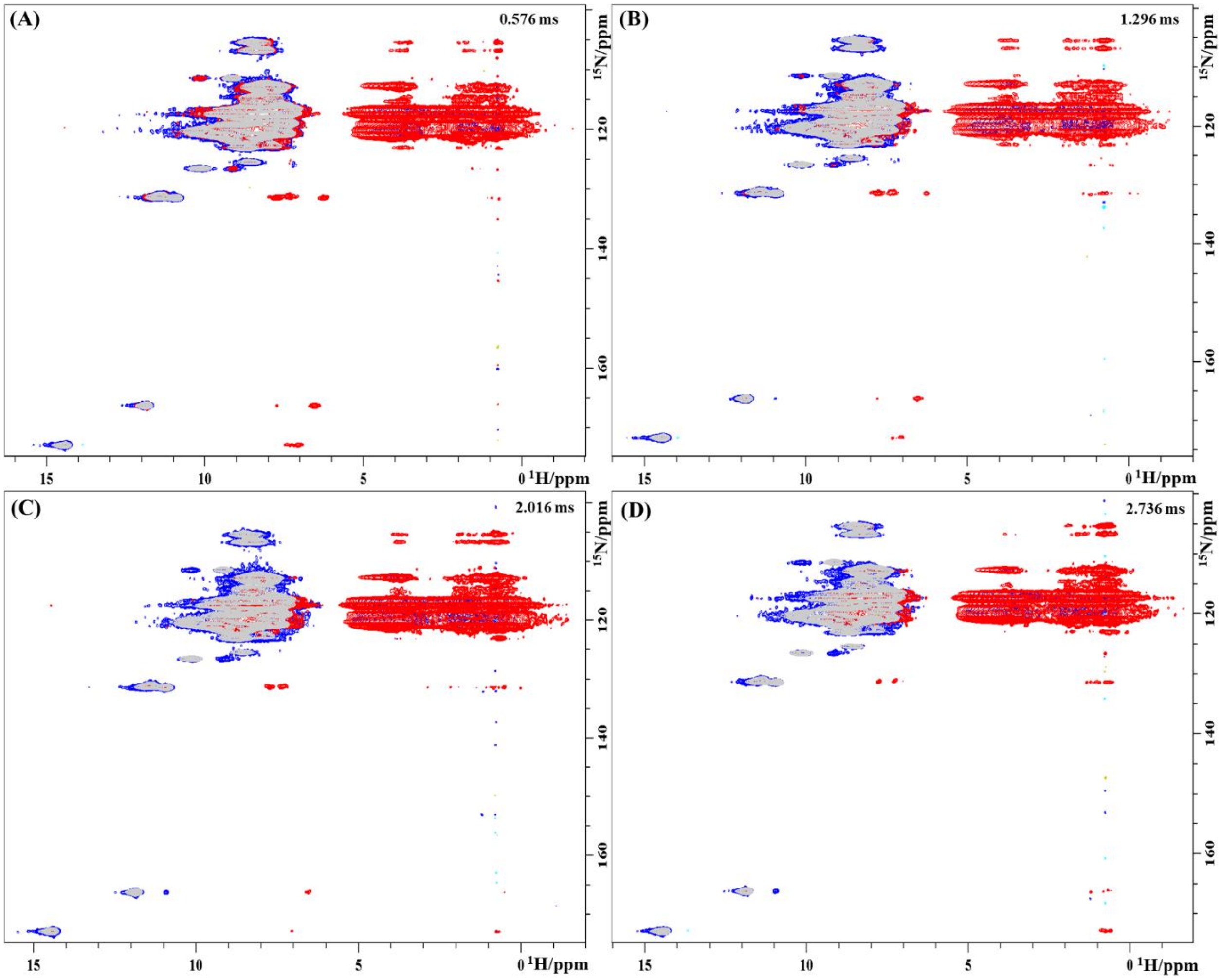

Figure S17 2D (H)N(H)H^{MODIST} (blue) and (H)N(H)H^{RFDR} (red) spectra of the influenza M2 protein as a function of mixing time (in ms): (A) 0.576, (B) 1.296, (C) 2.016, and (D) 2.736. The (H)NH reference spectrum is shown in grey. For all experiments the proton carrier frequency was set to 8.2 ppm. 55.555 kHz MAS was used. An 800 MHz spectrometer was used. Further experimental details of all experiments are provided in the experimental methods, below.

M2, 600 MHz

Figure S18 compares 2D (H)N(H)^{MODIST} (blue), (H)N(H)^{SPR5_4} (red) spectra under 4 different mixing times.

Figure S19-S20 compares 2D (H)N(H)H^{MODIST} (Figure S19) and SPR5$_4$ / π/4-SPR4$_2$ (Figure S20) experiments with different position of the carrier frequency.

Figure S21 shows two ^{15}N-^{15}N projections of the (H)N(H)(H)NH^{MODIST} spectra with two different mixing times.

Figure S22 compares ^{15}N-^{15}N projections: a (H)N(H)(H)NH^{MODIST} spectrum with a (H)N(H)(H)NH^{SPR5_4} spectrum.

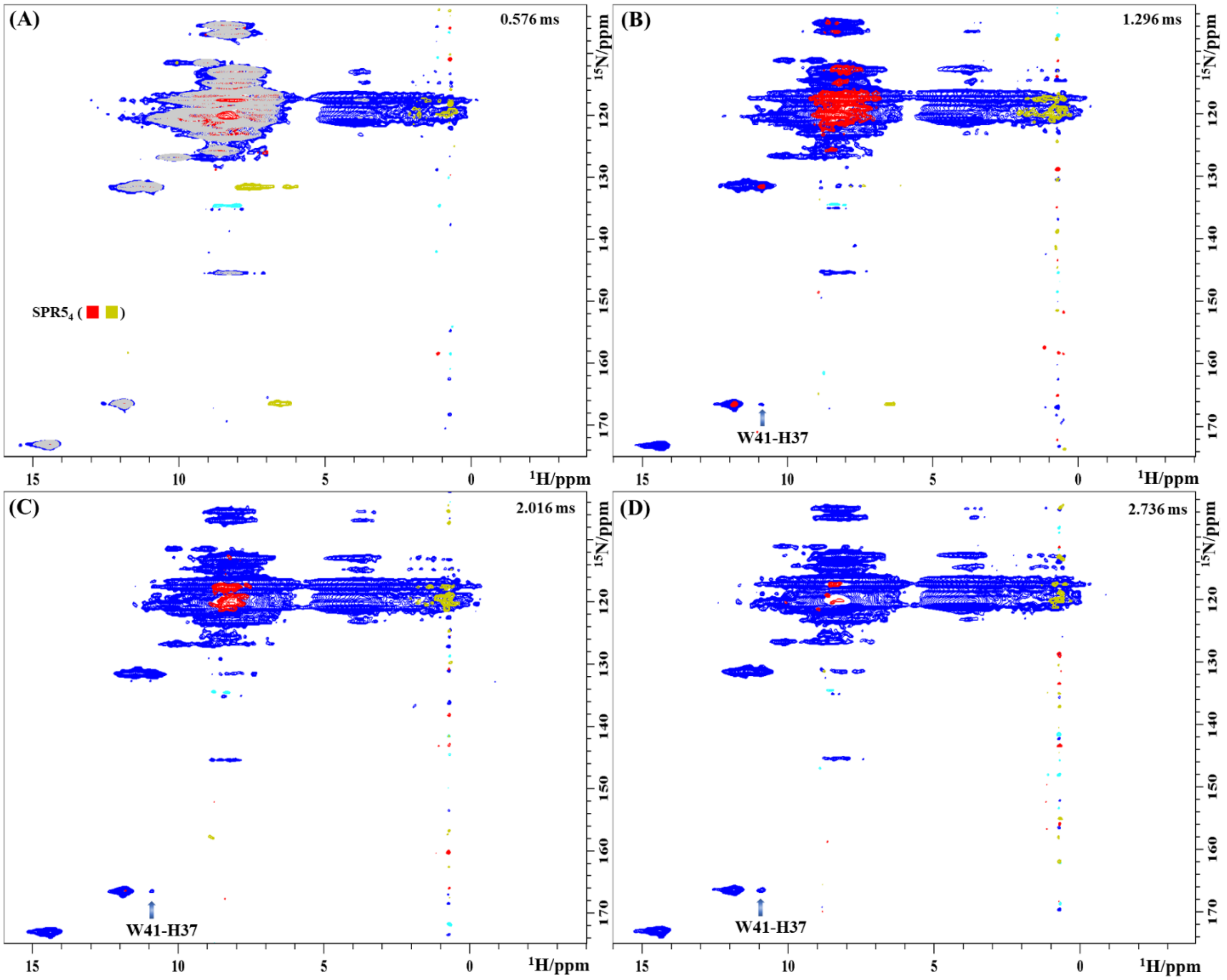

Figure S18 2D (H)N(H)H^{MODIST} (blue) and (H)N(H)H^{SPR5_4} (red (positive) / green (negative)) spectra of the influenza A M2 protein as a function of mixing time (in ms): 0.576, 1.296, 2.016, 2.736. The (H)NH reference spectrum is shown in gray. For all spectra, the proton carrier frequency was set to 9.5 ppm. 55.555 kHz MAS was used. Note that comparison of the total signal for double quantum sequences such as SPR5$_4$ is not meaningful, since direct transfer

results in negative cross-peaks and the potential for signal cancellation. The experimental details of all experiments are provided in the experimental methods, below.

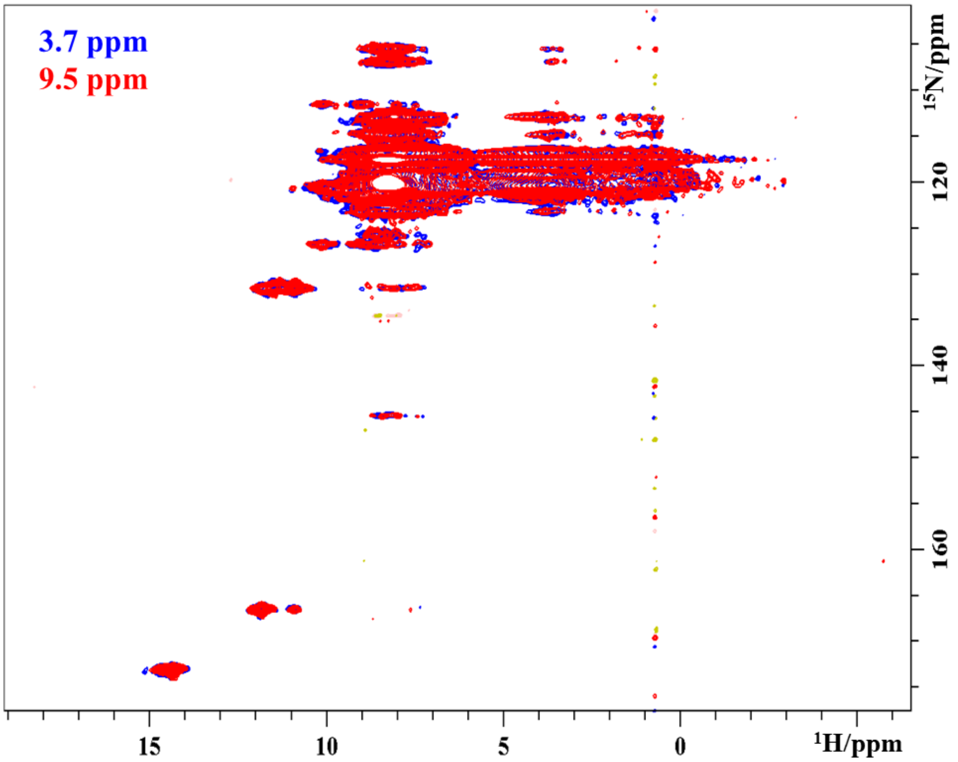

Figure S19 2D (H)N(H)H^{MODIST} spectra (2.736 ms mixing) with the carrier set to 9.5 ppm (red) or 3.7 ppm (blue). 55.555 kHz MAS was used. The experimental details of all experiments are provided in the experimental methods, below.

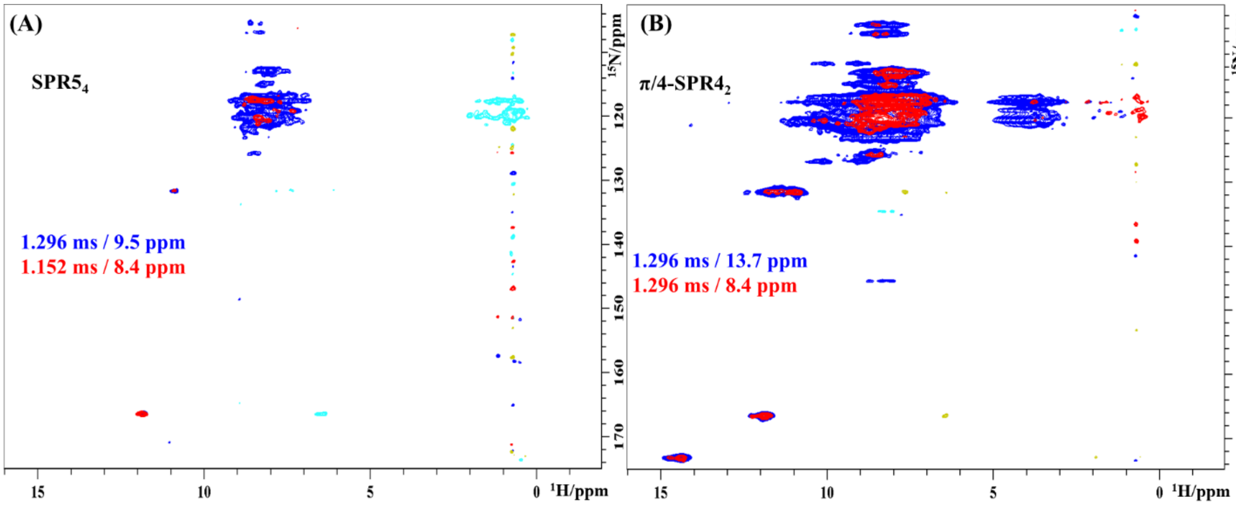

Figure S20 2D (H)N(H)H spectra with SPR5$_4$ (A) and π/4-SPR4$_2$ (B) mixing at two carrier frequency (CF) values. (A) Blue (positive) / cyan (negative) – 1.296 ms mixing and 9.5 ppm CF. Red (positive) / green (negative) – 1.152 ms

mixing and 8.4 ppm CF. (B) Blue (positive) / cyan (negative) – 1.296 ms mixing and 13.7 ppm CF. Red (positive) / green (negative) – 1.296 ms mixing and 8.4 ppm CF. 55.555 kHz MAS was used. Performance is best with the CF at 13.7 ppm (the case chosed for the main text comparison in figure 4. Note that comparison of the total signal for double quantum sequences such as SPR5_4 is not meaningful, since direct transfer results in negative cross-peaks and the potential for signal cancellation. Experimental details of all experiments are provided in the experimental methods, below.

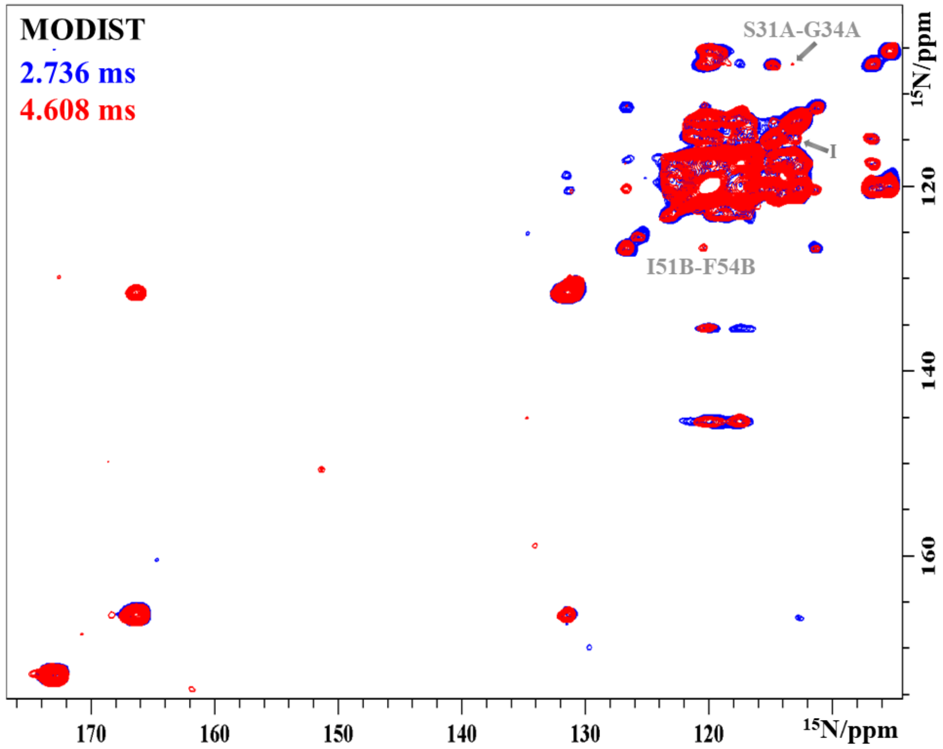

Figure S21 ^{15}N – ^{15}N projections of 3D (H)N(H)(H)NH^{MODIST} spectra with 2.736 ms (blue) and 4.608 ms (red) mixing. The proton carrier frequency was set to 8.4 ppm. The MAS was set to 55.555 kHz. Assigned correlations observed with longer mixing are labeled in gray. The roman letter 'I' indicates an unassigned correlation appearing with longer mixing. Experimental details of all experiments are provided in the experimental methods, below.

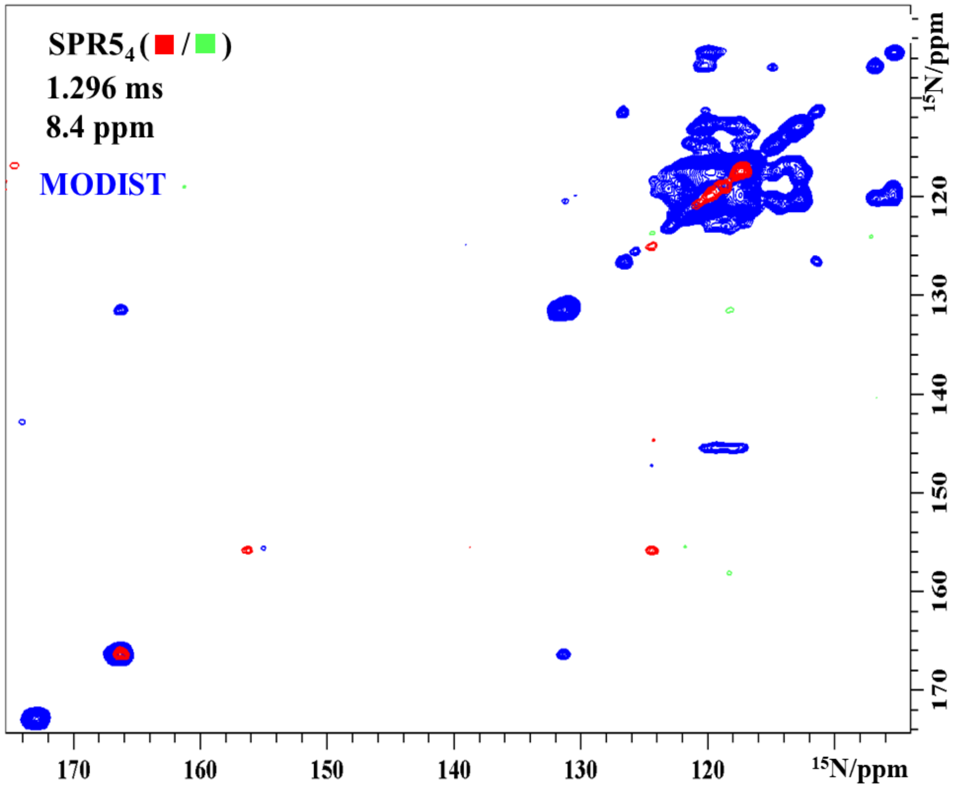

Figure S22 ^{15}N – ^{15}N projection of the 3D (H)N(H)(H)NH^{SPR5_4} (red (positive) / green (negative); 1.296 ms mixing) and (H)N(H)(H)NH^{MODIST} (blue; 2.736 ms mixing) spectra. The low intensity of SPR5$_4$ might be partly explained by the double quantum mixing: direct cross-peaks are negative, while relayed transfer is positive when relayed through a single intermediate spin. This can lead to cancellation of signal. The proton carrier frequency was set to 8.4 ppm. 55.555 kHz MAS was used. Experimental details of all experiments are provided in the experimental methods, below. MODIST and SP5$_4$ data were each recorded with 4 scans per point, and identical sweepwidths.

EXPERIMENTAL METHODS

Simulations

MODIST, SPR simulations were performed using in-house MATLAB scripts with the numerical solution of the equation of motion.[10]

Sample Preparation

Fully protonated microcrystalline chicken alpha-spectrin SH3 was prepared according to the protocols in the next references[11,12] and influenza A M2 protein, residues 18-60, was prepared

according to the protocols in the references [13,14]. Deuterated alpha-spectrin SH3 microcrystals were prepared according to the published protocols[5,15]. Each sample was packed into a Bruker 1.3 mm rotor via centrifugation.

Solid state NMR spectroscopy

The rf-field power of MODIST pulses was optimized using a single pulse calibration, detected in the 1D (H)NH spectrum. The width of the first proton pulse was set to one rotor period of duration (for example, 18 μs for 55.555 kHz MAS). The optimal rf-field power was obtained for zero (H)NH signal (180°-pulse).

Figure S23A shows 2D (H)N(H)H pulse sequences used with either RFDR, SPR, MODIST, or BASS-SD dipolar recoupling. The RFDR block consists of a train of π-pulses with XY-8 phase cycling[16] (with the exception of the data of Figure S10, for which fully protonated SH3 and 600 MHz spectrometer $XY4^{1}_{4}$ phase cycling[4] was used). SPR pulses are described in the 'Simulations' section. The BASS-SD pulse is a low-amplitude spin lock pulse with a tangential shape.[17]

Figure S23B shows 3D (H)N(H)(H)NH pulse sequences used with SPR, BASS-SD, and MODIST.

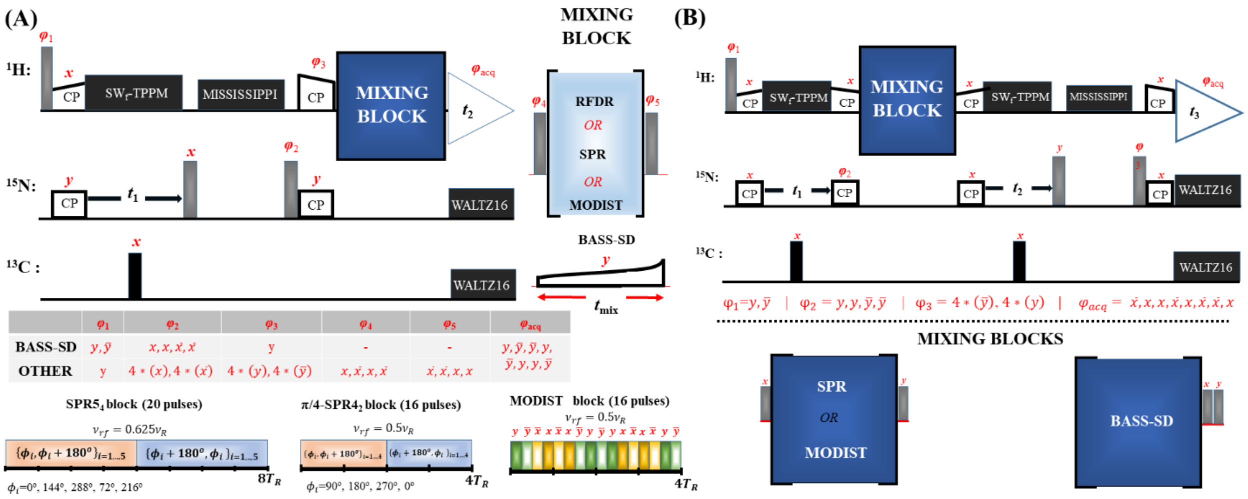

Figure S23 2D (H)N(H)H (A) and 3D (H)N(H)(H)NH (B) sequences. π/2-pulses are indicated by light, π-pulses by dark grey rectangles. All phase cycling is shown in Figure. The ramped CP transfers from proton to nitrogen as well as from nitrogen to proton in are indicated with constant power on the nitrogen channel and a ramp in power on the proton channel. During the inderect dimensions (t_1) and (t_2), SW_f-TPPM decoupling[18] is applied. A single π-pulse in the middle of t_1 and t_2 decouples carbon-nitrogen interactions. Water supression is implemented with the MISSISSIPI sequence.[19] During acquisition, WALTZ16 decoupling[20] is applied on nitrogen and carbon channels. RFDR: the RFDR block (eight rotor periods) consists of eight π-pulses every rotor period with XY8 phase cycling.[16] BASS-SD: the tan amplitude modulated pulse[17] has a constant phase. $SPR5_4$: the $SPR5_4$ block (eight rotor periods) consists of 20 0.5π-pulses with phase cycling: 0°, 180°, 144°, 324°, 288°, 108°, 72°, 252°, 216°, 36°, 180°, 0°, 324°, 144°, 108°, 288°, 252°, 72°, 36°, 216°, according to the next reference.[1] π/4-$SPR4_2$: the $SPR4_2$ block (four rotor periods) consists of 16 0.25π-pulses with phase cycling: 90°, 270°, 180°, 0°, 270°, 90°, 0°, 180°, 270°, 90°, 0°, 180°, 90°, 270°, 180°, 0°. MODIST: the MODIST block (four rotor periods) contains 16 π/4-pulses with phase cycling: 90°, 270°, 180°, 0°, 180°, 0°, 270°, 90°, 270°, 90°, 0°, 180°, 0°, 180°, 90°, 270°.

800 MHz: 2D (H)N(H)H and 3D (H)N(H)(H)NH experiments were acquired on a Bruker Avance III HD spectrometer operating at 18.8 T (800 MHz ^{1}H field strength), equipped with a 1.3 mm HCN MAS probe at 55.555 kHz MAS. The temperature of the nitrogen cooling gas was set to 235 K and 1000 to 1300 liters per hour. Heteronuclear dipolar interactions were decoupled with SW_f-TPPM,[18] WALTZ-16,[20] for water suppression MISSISSIPPI[19] was used. In all figures

below: SW – spectral width; TD – the size of FID; IN_F – an increment time; AQ – the acquisition time. 2 Dummy Scans were used.

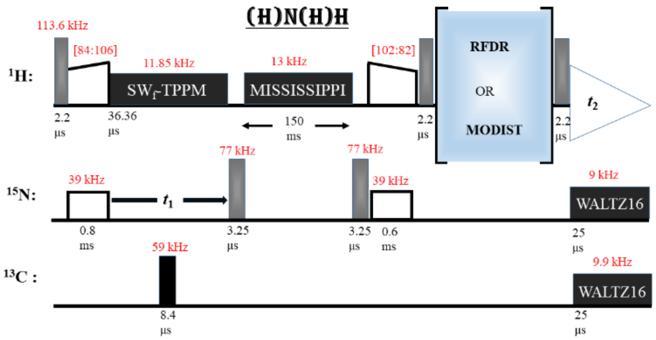

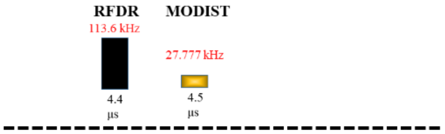

	¹H	¹⁵N
TD	2048	320
SW(ppm)	30.0923	30
IN_F(µs)		411.76
AQ(s)	0.0425984	0.0658815

Number Scans: 8 | Recycle delay: 2 s
Total Time of the experiment: 1.59 hours

Figure S24 The 2D (H)N(H)H pulse sequence and the experimental parameters used for SH3. Red numbers represent the rf-field power in kHz. Rectangle brackets indicate the minimal and maximal rf-field of ramped CPs. The width of hard pulses are in µs, while the total duration of CP and decoupling are in ms. RFDR and MODIST were used for dipolar recoupling.

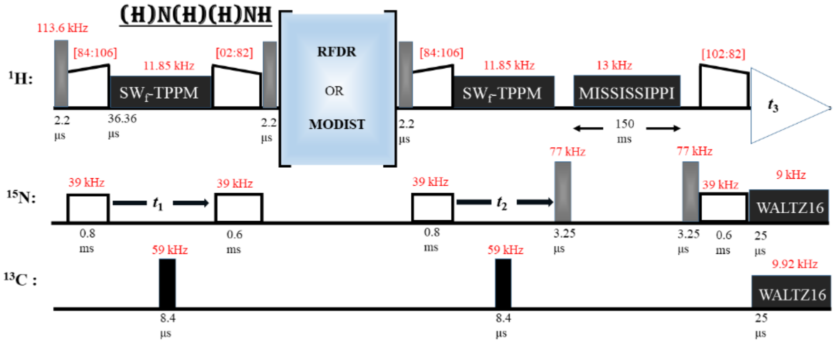

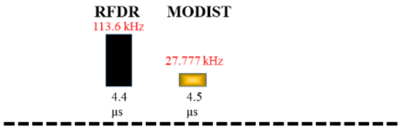

	¹H	¹⁵N	¹⁵N
TD	2048	84	84
SW(ppm)	30.0923	30	30
IN_F(µs)		411.76	411.76
AQ(s)	0.0425984	0.0172939	0.0172939

Number Scans: 16 | Recycle delay: 2 s |
Total Time of the experiment: 2.898 days

Figure S25 The 3D (H)N(H)(H)NH pulse seuqnce and the experimental parameters used for measurement on SH3. Red numbers represent the rf-field power in kHz. Rectangle brackets indicate the minimal and maximal rf-field of the ramped CPs. The width of hard pulses are in µs, while the total duration of CP and decoupling are in ms. RFDR and MODIST were used for dipolar recoupling.

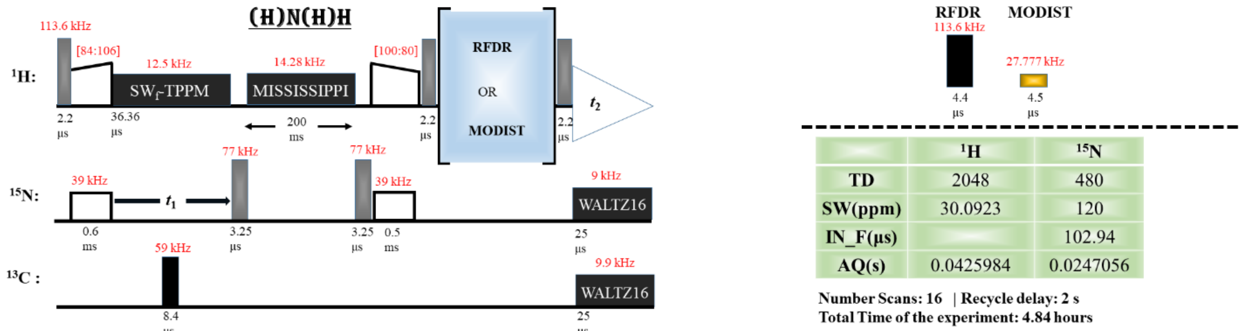

	^1H	^{15}N
TD	2048	480
SW(ppm)	30.0923	120
IN_F(μs)		102.94
AQ(s)	0.0425984	0.0247056

Number Scans: 16 | Recycle delay: 2 s
Total Time of the experiment: 4.84 hours

Figure S26 2D (H)N(H)H pulse sequence with the experimental parameters used for measurement on influenza A M2. Red numbers represent rf-field power in kHz. Rectangle brackets indicate the minimal and maximal rf-field of ramped CPs. The width of the hard pulses are in μs, while total widths of CP and decoupling blocks are in ms. RFDR and MODIST were used for dipolar recoupling.

600 MHz: 2D (H)N(H)H, 3D (H)N(H)(H)NH and (H)C(H)(H)CH experiments were acquired on a Bruker Avance III HD spectrometer operating at 14.1 T (600 MHz ^1H frequency) using a DVT600W2 BL1.3 mm HXY probe. The experiments were performed at 55.555 kHz MAS, the temperature of the nitrogen cooling gas set to 243 K, and 1000 to 1300 liters per hour. For decoupling of the heteronuclear dipolar interactions SW$_f$-TPPM,[18] was used on the proton channel, and WALTZ-16[20] was used on heteronuclear channels. MISSISSIPPI[19] water suppression was applied. In all Figures below: SW – spectral width; TD – the size of FID; IN_F – an increment time; AQ – the acquisition time. 4 Dummy Scans were used.

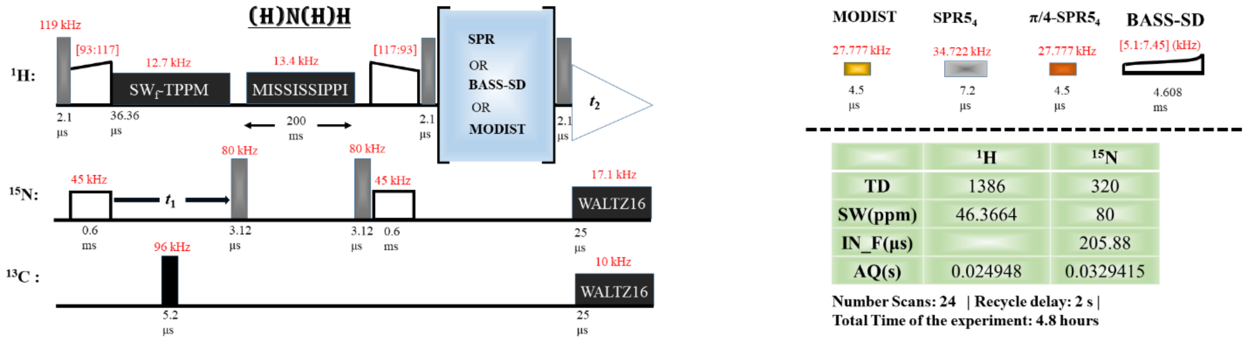

	^1H	^{15}N
TD	1386	320
SW(ppm)	46.3664	80
IN_F(μs)		205.88
AQ(s)	0.024948	0.0329415

Number Scans: 24 | Recycle delay: 2 s |
Total Time of the experiment: 4.8 hours

Figure S27 The 2D (H)N(H)H spectrum and the experimental parameters used for measurement on influenza A M2. Red numbers represent rf-field power in kHz. Rectangle brackets indicate the minimal and maximal rf-field of ramped CPs. The width of the hard pulses are in µs, while total duration of CP and decoupling are in ms. SPR5$_4$, π/4-SPR4$_2$, BASS-SD, and MODIST were used for dipolar recoupling. For BASS-SD, the two π/2-pulses next to the mixing block were omitted.

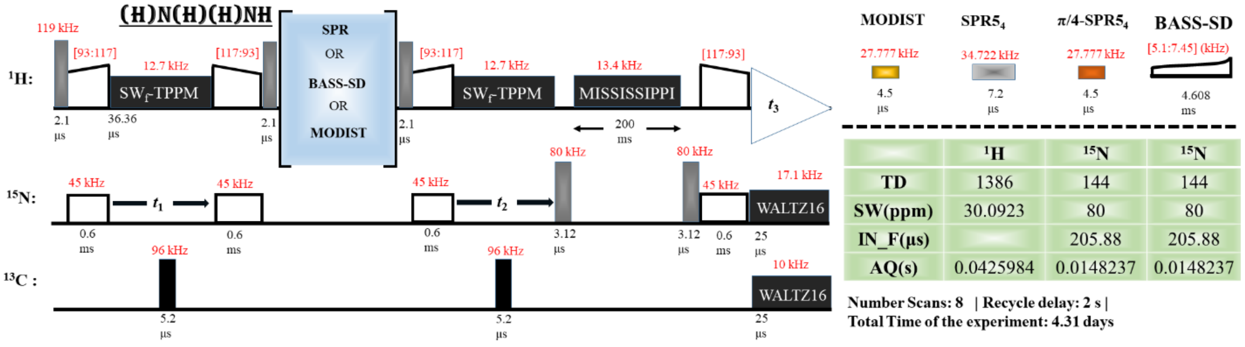

Figure S28 The 3D (H)N(H)(H)NH pulse sequence and the experimental parameters used for measuring influenza A M2. Red numbers represent rf-field power in kHz. Rectangle brackets indicate the minimal and maximal rf-field of ramped CPs. The width of the hard pulses are in µs, while total duration of CP and decoupling are in ms. SPR5$_4$, π/4-SPR4$_2$, BASS-SD, and MODIST were used for dipolar recoupling.

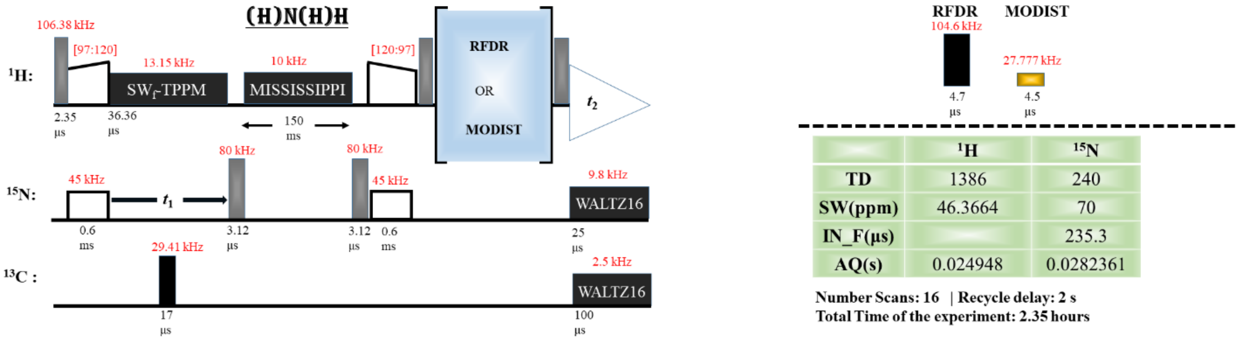

Figure S29 2D (H)N(H)H pulse sequence with the experimental parameters used for measurement on fully protonated SH3. Red numbers represent rf-field power in kHz. Rectangle brackets indicate the minimal and maximal rf-field of ramped CPs. The width of the hard pulses are in µs, while total widths of CP and decoupling blocks are in ms. RFDR and MODIST were used for dipolar recoupling.

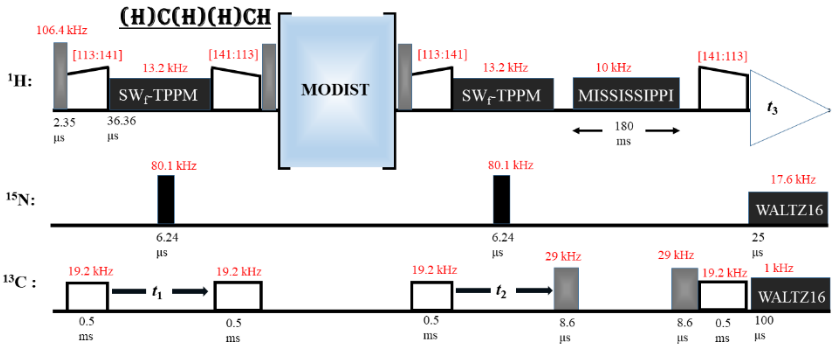

	¹H	¹³C	¹³C
TD	1386	270	512
SW(ppm)	46.3664	100	176
IN_F(µs)		66.38	37.72
AQ(s)	0.024948	0.0089613	0.0096553

Figure S30 The 3D (H)C(H)(H)CH pulse sequence and the experimental parameters used for measuring fully protonated SH3. Red numbers represent rf-field power in kHz. Rectangle brackets indicate the minimal and maximal rf-field of ramped CPs. The width of the hard pulses are in µs, while total duration of CP and decoupling are in ms. MODIST was used for dipolar recoupling.

1200 MHz: 3D (H)N(H)(H)NH spectra were acquired on a Bruker Avance NEO spectrometer operating at 28.18 T (1200 MHz ^{1}H frequency) using a 1.3 mm HCN probe. The experiments were performed at 55.555 kHz MAS, the temperature of the nitrogen cooling gas set to 245 K using 1000 liters per hour of flow. For decoupling of the heteronuclear dipolar interactions and water suppression SW$_f$-TPPM,[18] WALTZ-16[20] and MISSISSIPPI[19] were applied. In the Figure below: SW – spectral width; TD – the size of FID; IN_F – an increment time; AQ – the acquisition time. 4 Dummy Scans were used.

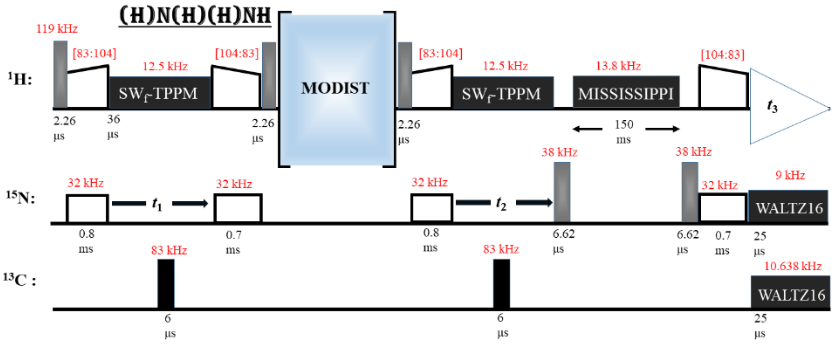

	¹H	¹⁵N	¹⁵N
TD	2048	120	120
SW(ppm)	40.4434	35	35
IN_F(µs)		234.8895	234.8895
AQ(s)	0.0210944	0.0140934	0.0140934

Figure S31 The 3D (H)N(H)(H)NH experiment with the experimental parameters used for measuring fully protonated SH3. Red numbers represent rf-field power in kHz. Rectangle brackets indicate the minimal and maximal

rf-field of ramped CPs. The width of the hard pulses are in µs, while total duration of CP and decoupling are in ms. MODIST was used for dipolar recoupling.

850 MHz: 2D (H)N(H)H and 3D (H)N(H)(H)NH experiments were acquired on a Bruker Avance III spectrometer operating at 19.97 T (850 MHz ^{1}H field strength), equipped with a 1.3 mm HCN MAS probe at 55.555 kHz MAS. The temperature of the nitrogen cooling gas was set to 245 K. Heteronuclear dipolar interactions were decoupled with SW_f-TPPM,[18] (proton channel) and WALTZ-16,[20] (nitrogen channel). For water suppression, MISSISSIPPI[19] was used. In all figures below: SW – spectral width; TD – the size of FID; IN_F – an increment time; AQ – the acquisition time. 4 Dummy Scans were used.

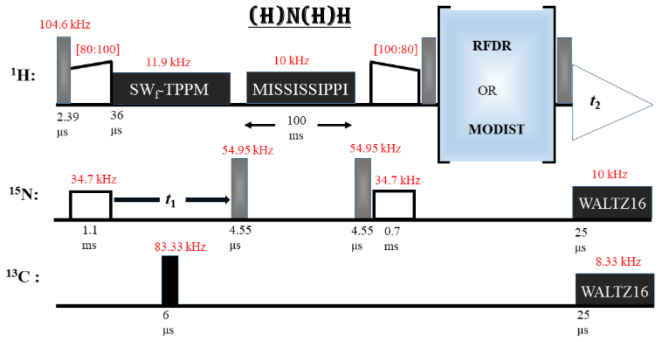

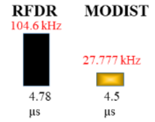

	¹H	¹⁵N
TD	2330	260
SW(ppm)	30.4694	35
IN_F(µs)		331.59
AQ(s)	0.044969	0.0431065

Number Scans: 16 | Recycle delay: 2 s
Total Time of the experiment: 2.58 hours

Figure S32 2D (H)N(H)H pulse sequence with the experimental parameters used for measurement on deuterated SH3. Red numbers represent rf-field power in kHz. Rectangle brackets indicate the minimal and maximal rf-field of ramped CPs. The width of the hard pulses are in µs, while total widths of CP and decoupling blocks are in ms. RFDR and MODIST were used for dipolar recoupling.

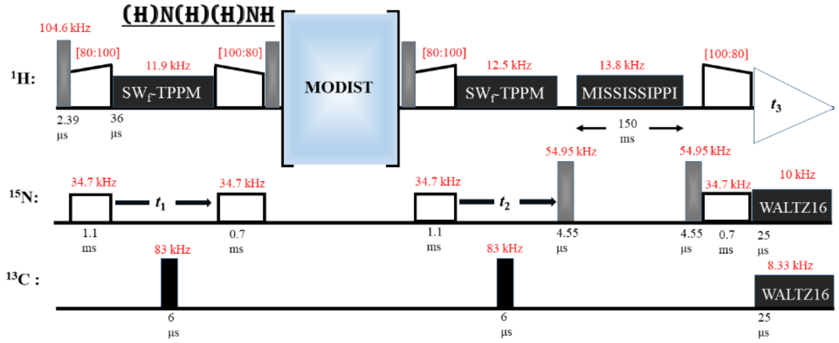

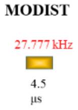

	¹H	¹⁵N	¹⁵N
TD	2330	128	128
SW(ppm)	30.4694	30	30
IN_F(µs)		386.8536	386.8536
AQ(s)	0.044969	0.0247586	0.0247586

Number Scans: 8 | Recycle delay: 2 s |
Total Time of the experiment: 3.345 days

Figure S33 The 3D (H)N(H)(H)NH experiment with the experimental parameters used for measuring deuterated SH3. Red numbers represent rf-field power in kHz. Rectangle brackets indicate the minimal and maximal rf-field of ramped CPs. The width of the hard pulses are in µs, while total duration of CP and decoupling are in ms. MODIST was used for dipolar recoupling.

950 MHz: The 2D (H)N(H)H spectrum was acquired on a Bruker Avance III HD spectrometer operating at 22.3 T (950 MHz ^{1}H field strength), equipped with a 0.7 mm HCDN MAS probe at 100 kHz MAS. The temperature of the nitrogen cooling gas was set to 260 K with 400 liters per hour. Heteronuclear dipolar interactions were decoupled with SW_f-TPPM,[18] (proton channel) and WALTZ-16,[20] (nitrogen channel). For water suppression, MISSISSIPPI[19] was used. In all figures below: SW – spectral width; TD – the size of FID; IN_F – an increment time; AQ – the acquisition time. 4 Dummy Scans were used.

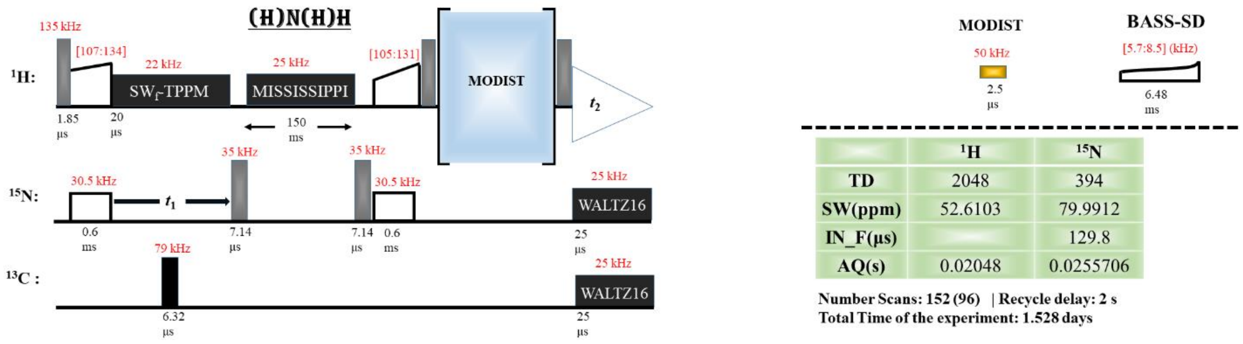

	¹H	¹⁵N
TD	2048	394
SW(ppm)	52.6103	79.9912
IN_F(µs)		129.8
AQ(s)	0.02048	0.0255706

Figure S34 The 2D (H)N(H)H pulse sequence and the experimental parameters used for SH3. Red numbers represent the rf-field power in kHz. Rectangle brackets indicate the minimal and maximal rf-field of ramped CPs. The width of hard pulses are in µs, while the total duration of CP and decoupling are in ms. MODIST and BASS-SD were used for dipolar recoupling. For Figure S10A and B 152 and 96 scans were used, respectively.

BRUKER PULSE PROGRAMS

The width of MODIST pulses is automatically calculated using the 'cnst31' parameter (the MAS rate in Hz). Note that there is currently no protection against long acquisitions that occur if this parameter is mistakenly set too low. Use only at your own risk.

2D (H)N(H)H MODIST

```
;1H-detected and CP based hNH experiment with MODIST mixing time
;with 15N and 13C decoupling
;parameters:
;p1 : 1H 90 pulse @ plw1
;pl1 : 1H power for 90 pulse
;p15 : contact time for H->N CP
;pl2 : 15N power for H->N CP
;sp0 : 1H power for H->N CP
;spnam0 : Ramp90.100
;spoffs0 : 0


;p7 : 15N 90 pulse @ plw7
;pl7 : 15N power for 90 pulse
;pl8 :15N power for 180 pulse

;pl11 : MODIST 0.5*MAS rate
;l21 : Number of MODIST blocks

;p3 : 13C 90 pulse @ plw3
;pl3 : 13C power for 90 pulse

;cpdprg1 : 1H decoupling (sltppm_40pTr41 for 1Hprot, waltz16_pl12 for 2Hprot)
;cpdprg2 : 15N decoupling (waltz16_pl16)
;cpdprg3 : 13C decoupling (waltz16_pl17)

;pl12 : 1H decoupling power for (tppm/waltz16)
;pl13: water suppression
;pl16: 15N decoupling power (waltz16)
;pl17: 13C decoupling power (waltz16)

;pcpd1 : 25u (waltz16 10 kHz) - 33.33 (sltppm 15kHz)
;pcpd4 : 33.33 (sltppm 15kHz)
;pcpd2 : 25u (waltz16 10 kHz)
;pcpd3 : 25u (waltz16 10 kHz)

;$CLASS=Solids
;$DIM=1D
;$TYPE=cross polarisation
;$OWNER=Bruker
prosol relations=<solids_cp>

#include <Avancesolids.incl>

;cnst19 : carrier offset for MODIST
```

```
;cnst21 : on resonance, usually = 0
;cnst11 : to adjust t=0 for acquisition, if digmod = baseopt
"acqt0=1u*cnst11"
"in0=inf1"

"p11 = ((1s/cnst31)/4)"

"l22=16"

define delay mix
"mix = (l21*(l22*p11))"

1 ze

2 d1 do:f2 do:f3
  mix
#include <p15_prot.incl>
#include <aq_prot.incl>

  (p1  pl1 ph3):f1
  (p15 pl2 ph2):f2 (p15:sp0 ph10):f1

  1u cpds1:f1

if "p3*2 > d0" goto RAWEVOL
   (center (d0) (p3*2 ph0 pl3):f3)
if "p3*2 <= d0" goto DECOFF

RAWEVOL, 1u
  d0
DECOFF,  1u do:f1

;;;;;;;;;;;;;;; water suppression block starts
  (p7 pl7 ph5):f2
  (p30*0.25  pl13 ph0):f1
  (p30*0.25  pl13 ph1):f1
  (p30*0.25  pl13 ph0):f1
  (p30*0.25  pl13 ph1):f1

  (p7 pl7 ph6):f2
;;;;;;;;;;;;;;; water suppression block ends

  (p17 pl20 ph7):f2 (p17:sp10 ph11):f1


(p1 pl1 ph4):f1
```

```
;;;;;;;;;;;;;cnst19 is used to put proton carrier frequency during MODIST
1u fq=cnst19(ppm):f1
 ;;;;;;;;;;;;;;; MODIST block starts
3
4 (p11 pl11 ph8^):f1
  lo to 4 times 16
  lo to 3 times l21
;;;;;;;;;;;;;;; MODIST block ends
  1u fq=cnst21(ppm):f1

  1u cpds2:f2 cpds3:f3
  (p1 pl1 ph14):f1
  go=2 ph31
  1m do:f2 do:f3

  10m mc #0 to 2
  F1PH(calph(ph2,+90) , caldel(d0,+in0))

HaltAcqu, 1m      ;jump address for protection files
exit                    ;quit

ph0=0
ph1=1
ph3  = 1
ph10 = 0
ph2 =  1
ph5 =  0
ph6 =  0 0 0 0 2 2 2 2
ph12 = 1 1 1 1
ph7 =  1
ph8 = 1 3 2 0 2 0 3 1 3 1 0 2 0 2 1 3
ph4=0 2 0 2
ph14= 2 2 0 0
ph11 = 1 1 1 1
ph31 = 1 3 3 1 3 1 1 3
```

3D (H)N(H)(H)NH MODIST

```
;can be run as 4D HN(H)(H)NH
;parameters:
;p1 : 1H 90 pulse @ plw1
;pl3 : 13C power for 180
;p3 : 90 pulse at pl3
;p15 : contact time at pl1 (f1) and pl2 (f2)
;pl1 : power for p1
;sp0 : proton power level during contact
;pl2 : =120dB, not used
;pl12 : decoupling power level (if not pl13)
```

```
;pl13 : special decoupling power level
;d1 : recycle delay
;cnst21 : on resonance, usually = 0
;pcpd2 : pulse length in decoupling sequence
;cpdprg2 : cw, tppm (at pl12), or lgs, cwlg. cwlgs (LG-decoupling
;spnam0 : use e.g. ramp.100 for variable amplitude CP
;here pl13 is used instead of pl12)
;zgoptns : -Dfslg, -Dlacq, or blank
;pl11 : MODIST 0.5*MAS rate
;l21 : Number of MODIST blocks
;$CLASS=Solids
;$TYPE=cross polarisation
;$OWNER=Bruker
prosol relations=<solids_cp>

#include <Avancesolids.incl>
;cnst19 : carrier offset for MODIST
;cnst21 : on resonance, usually = 0
;cnst11 : to adjust t=0 for acquisition, if digmod = baseopt
"acqt0=1u*cnst11"
"in0=inf1"
"in30=inf1"
"in10=inf2"
"in20=inf3"
"d0=0"
"d10=0"
"d20=0"
;aqseq 321

"d31=1s/cnst31"

"p11 = (d31/4)"
"l22=16"

define delay mix
"mix = (l21*(l22*p11))"

1 ze
  mix
  1u fq=cnst21:f1
2 d1 do:f2 do:f3

  (p1  pl1 ph7):f1
  (center (d0) (p7*2 ph0 pl7):f2 (p3*2 ph0 pl3):f3)
  (p15 pl2 ph2):f2 (p15:sp0 ph10):f1
  1u cpds1:f1
```

```
  (center (d10) (p3*2 ph0 pl3):f3)
  1u do:f1
  (p17 pl20 ph4):f2 (p17:sp10 ph3):f1

  (p1  pl1 ph5):f1
 ;;;;;;;;;;;;;;cnst19 is used to put proton carrier frequency during MODIST
1u fq=cnst19(ppm):f1
 ;;;;;;;;;;;;;;;; MODIST block starts
3
4 (p11 pl11 ph8^):f1
  lo to 4 times l22
  lo to 3 times l21
;;;;;;;;;;;;;;;; MODIST block ends
  1u fq=cnst21(ppm):f1

 (p1  pl1 ph6):f1

  (p15 pl2 ph12):f2 (p15:sp0 ph20):f1
  1u cpds1:f1
   (center (d20) (p3*2 ph0 pl3):f3)
  1u do:f1

  (p7 pl7 ph15):f2
  1u fq=cnst28:f1
 (p30*0.25 pl13 ph0):f1
 (p30*0.25 pl13 ph1):f1
 (p30*0.25 pl13 ph0):f1
 (p30*0.25 pl13 ph1):f1

  1u fq=cnst21:f1
  (p7 pl7 ph16):f2

  (p17 pl20 ph13):f2 (p17:sp10 ph14):f1

  1u cpds2:f2 cpds3:f3

  go=2 ph31
  1m do:f2     do:f3
  10m mc #0 to 2
  F1PH(calph(ph10, -90), caldel(d0, +in0))  ;proton dim
  F2PH(calph(ph2, +90), caldel(d10, +in10))   ;first 15N dim
  F3PH(calph(ph12, +90), caldel(d20, +in20))  ;second 15N dim


HaltAcqu, 1m      ;jump address for protection files
exit                    ;quit
```

```
ph0=0
ph1=1

ph7  = 1 3
ph3  = 1 1 3 3
ph6  = 1 1 1 1
ph8 = 1 3 2 0 2 0 3 1 3 1 0 2 0 2 1 3
ph16 = 1 1 1 1 3 3 3 3
ph10 = 0
ph2 =  0
ph4 =  0
ph5 =  0
ph12 = 0
ph20 = 0
ph15 = 1
ph13 = 0
ph14 = 0
ph31 = 0 2 2 0 2 0 0 2
ph18 = 0 1 0 1 1 0 1 0
```

REFERENCE

(1) Zhang, Z.; Oss, A.; Org, M.-L.; Samoson, A.; Li, M.; Tan, H.; Su, Y.; Yang, J. Selectively Enhanced 1H–1H Correlations in Proton-Detected Solid-State NMR under Ultrafast MAS Conditions. *J. Phys. Chem. Lett.* **2020**, *11* (19), 8077–8083.

(2) Xiao, H.; Zhang, Z.; Yang, J. Theory of Frequency-Selective Homonuclear Dipolar Recoupling in Solid-State NMR. *J. Chem. Phys.* **2021**.

(3) Xiao, H.; Zhang, Z.; Zhao, Y.; Yang, J. Spectral Editing of Alanine, Serine, and Threonine in Uniformly Labeled Proteins Based on Frequency-Selective Homonuclear Recoupling in Solid-State NMR. *J. Biomol. NMR* **2021**, *75* (4), 193–202.

(4) Nishiyama, Y.; Zhang, R.; Ramamoorthy, A. Finite-Pulse Radio Frequency Driven Recoupling with Phase Cycling for 2D (1)H/(1)H Correlation at Ultrafast MAS Frequencies. *J. Magn. Reson. San Diego Calif 1997* **2014**, *243*, 25–32.

(5) Chevelkov, V.; Rehbein, K.; Diehl, A.; Reif, B. Ultrahigh Resolution in Proton Solid-State NMR Spectroscopy at High Levels of Deuteration. *Angew. Chem. Int. Ed.* **2006**, *45* (23), 3878–3881.

(6) van Rossum, B.-J.; Castellani, F.; Pauli, J.; Rehbein, K.; Hollander, J.; de Groot, H. J. M.; Oschkinat, H. Assignment of Amide Proton Signals by Combined Evaluation of HN, NN and HNCA MAS-NMR Correlation Spectra. *J. Biomol. NMR* **2003**, *25* (3), 217–223.

(7) Linser, R.; Fink, U.; Reif, B. Assignment of Dynamic Regions in Biological Solids Enabled by Spin-State Selective NMR Experiments. *J. Am. Chem. Soc.* **2010**, *132* (26), 8891–8893.

(8) van Rossum, B.-J.; Castellani, F.; Rehbein, K.; Pauli, J.; Oschkinat, H. Assignment of the Nonexchanging Protons of the α-Spectrin SH3 Domain by Two- and Three- Dimensional 1H–13C Solid-State Magic-Angle Spinning NMR and Comparison of Solution and Solid-State Proton Chemical Shifts. *ChemBioChem* **2001**, *2* (12), 906–914.

(9) Pauli, J.; Baldus, M.; van Rossum, B.; de Groot, H.; Oschkinat, H. Backbone and Side-Chain 13C and 15N Signal Assignments of the α-Spectrin SH3 Domain by Magic Angle Spinning Solid-State NMR at 17.6 Tesla. *ChemBioChem* **2001**, *2* (4), 272–281.
(10) Nimerovsky, E.; Goldbourt, A. Insights into the Spin Dynamics of a Large Anisotropy Spin Subjected to Long-Pulse Irradiation under a Modified REDOR Experiment. *J. Magn. Reson.* **2012**, *225*, 130–141.
(11) Pauli, J.; van Rossum, B.; Förster, H.; de Groot, H. J. M.; Oschkinat, H. Sample Optimization and Identification of Signal Patterns of Amino Acid Side Chains in 2D RFDR Spectra of the α-Spectrin SH3 Domain. *J. Magn. Reson.* **2000**, *143* (2), 411–416.
(12) Chevelkov, V.; Faelber, K.; Schrey, A.; Rehbein, K.; Diehl, A.; Reif, B. Differential Line Broadening in MAS Solid-State NMR Due to Dynamic Interference. *J. Am. Chem. Soc.* **2007**, *129* (33), 10195–10200.
(13) Schnell, J. R.; Chou, J. J. Structure and Mechanism of the M2 Proton Channel of Influenza A Virus. *Nature* **2008**, *451* (7178), 591–595.
(14) Andreas, L. B.; Eddy, M. T.; Pielak, R. M.; Chou, J.; Griffin, R. G. Magic Angle Spinning NMR Investigation of Influenza A M218−60: Support for an Allosteric Mechanism of Inhibition. *J. Am. Chem. Soc.* **2010**, *132* (32), 10958–10960.
(15) Xue, K.; Mühlbauer, M.; Mamone, S.; Sarkar, R.; Reif, B. Accurate Determination of 1H-15N Dipolar Couplings Using Inaccurate Settings of the Magic Angle in Solid-State NMR Spectroscopy. *Angew. Chem. Int. Ed.* **2019**, *58* (13), 4286–4290.
(16) Bennett, A. E.; Rienstra, C. M.; Griffiths, J. M.; Zhen, W.; Lansbury, P. T.; Griffin, R. G. Homonuclear Radio Frequency-Driven Recoupling in Rotating Solids. *J. Chem. Phys.* **1998**, *108* (22), 9463–9479.
(17) Jain, M. G.; Lalli, D.; Stanek, J.; Gowda, C.; Prakash, S.; Schwarzer, T. S.; Schubeis, T.; Castiglione, K.; Andreas, L. B.; Madhu, P. K.; et al Selective 1H–1H Distance Restraints in Fully Protonated Proteins by Very Fast Magic-Angle Spinning Solid-State NMR. *J. Phys. Chem. Lett.* **2017**, *8* (11), 2399–2405.
(18) Thakur, R. S.; Kurur, N. D.; Madhu, P. K. Swept-Frequency Two-Pulse Phase Modulation for Heteronuclear Dipolar Decoupling in Solid-State NMR. *Chem. Phys. Lett.* **2006**, *426* (4), 459–463.
(19) Zhou, D. H.; Rienstra, C. M. High-Performance Solvent Suppression for Proton Detected Solid-State NMR. *J. Magn. Reson.* **2008**, *192* (1), 167–172.
(20) Shaka, A. J.; Keeler, J.; Frenkiel, T.; Freeman, R. An Improved Sequence for Broadband Decoupling: WALTZ-16. *J. Magn. Reson. 1969* **1983**, *52* (2), 335–338.
